# Real-world experience with circulating tumor DNA in cerebrospinal fluid from patients with central nervous system tumors

**DOI:** 10.1186/s40478-024-01846-4

**Published:** 2024-09-17

**Authors:** Richard A. Hickman, Alexandra M. Miller, Bridget M. Holle, Justin Jee, Si-Yang Liu, Dara Ross, Helena Yu, Gregory J. Riely, Christina Ombres, Alexandra N. Gewirtz, Anne S. Reiner, Subhiksha Nandakumar, Adam Price, Thomas J. Kaley, Maya S. Graham, Chad Vanderbilt, Satshil Rana, Katherine Hill, Kiana Chabot, Carl Campos, Khedoudja Nafa, Neerav Shukla, Matthias Karajannis, Bob Li, Michael Berger, Marc Ladanyi, Elena Pentsova, Adrienne Boire, A. Rose Brannon, Tejus Bale, Ingo K. Mellinghoff, Maria E. Arcila

**Affiliations:** 1 Human Oncology and Pathogenesis Program, Sloan Kettering Institute, New York, NY 10065 USA; 2 Department of Pathology, Memorial Sloan Kettering Cancer Center, 1275 York Ave., New York, NY 10065 USA; 3 Department of Neurology, Memorial Sloan Kettering Cancer Center, 1275 York Ave., New York, NY 10065 USA; 4 Department of Pediatrics, Memorial Sloan Kettering Cancer Center, New York, NY 10065 USA; 5 Department of Medicine, Memorial Sloan Kettering Cancer Center, New York, NY 10065 USA; 6 Department of Epidemiology and Biostatistics, Memorial Sloan Kettering Cancer Center, New York, NY 10065 USA; 7 Present Address: Foundation Medicine, Inc., 150 Second Street, Cambridge, MA 02141 USA; 8 Present Address: Department of Neurology, Perlmutter Cancer Center, NYU Langone Health and NYU Grossman School of Medicine, New York, NY 10016 USA

## Abstract

The characterization of genetic alterations in tumor samples has become standard practice for many human cancers to achieve more precise disease classification and guide the selection of targeted therapies. Cerebrospinal fluid (CSF) can serve as a source of tumor DNA in patients with central nervous system (CNS) cancer. We performed comprehensive profiling of CSF circulating tumor DNA (ctDNA) in 711 patients using an FDA-authorized platform (MSK-IMPACT™) in a hospital laboratory. We identified genetic alterations in 489/922 (53.0%) CSF samples with clinically documented CNS tumors. None of 85 CSF samples from patients without CNS tumors had detectable ctDNA. The distribution of clinically actionable somatic alterations was consistent with tumor-type specific alterations across the AACR GENIE cohort. Repeated CSF ctDNA examinations from the same patients identified clonal evolution and emergence of resistance mechanisms. ctDNA detection was associated with shortened overall survival following CSF collection. Next-generation sequencing of CSF, collected through a minimally invasive lumbar puncture in a routine hospital setting, provides clinically actionable cancer genotype information in a large fraction of patients with CNS tumors.

**Supplementary Information:**

The online version contains supplementary material available at 10.1186/s40478-024-01846-4.

## Introduction

Tumors affecting the central nervous system (CNS) are a heterogeneous group of neoplasms that pose distinct challenges in diagnosis, treatment, and monitoring [12]. CNS metastases occur in up to 40% of cancer patients and confer poor prognosis. As strategies to treat systemic cancer have improved, the CNS frequently represents a site of late disease recurrence with molecular features that can differ from peripheral metastatic sites [1]. Primary CNS tumors represent an equally heterogenous group of neoplasms with considerable disease and treatment-related morbidity and mortality [18].

The characterization of genetic alterations in tumor samples has become standard practice for many human cancers to achieve more precise disease classification and guide the selection of targeted therapies [8, 11, 14]. Genomic tumor profiling relies on the collection of tumor tissue which, in the case of CNS cancers, is obtained through a neurosurgical procedure. Several studies have demonstrated that circulating tumor DNA (ctDNA) can be found in cerebrospinal fluid (CSF) from patients with CNS cancer. In contrast, common plasma-based liquid biopsies are generally unsuccessful in detecting circulating tumor DNA (ctDNA) from CNS tumors, in part due to the blood–brain barrier [4–6, 13, 15, 19, 21].

CSF-based liquid biopsies have shown feasibility and superiority for ctDNA detection in small-scale studies, demonstrating the presence of tumor derived genetic variants at high levels without the dilutional effect imparted by cell free DNA derived from the hematopoietic compartment in the peripheral circulation. However, the feasibility and utility of performing CSF ctDNA profiling in a routine hospital setting is currently unknown, as are the pre-analytic factors that may influence sequencing success. We previously reported a pilot study in 53 patients describing the potential utility of CSF ctDNA sequencing in CSF [15]. We subsequently validated this test for routine clinical use and demonstrated that profiling of CSF cfDNA is superior to profiling of CSF genomic DNA (gDNA) in capturing mutations at high variant allele frequency [4]. Here we report our “real-world” experience using this clinically validated, FDA-authorized platform, MSK-IMPACT™, to routinely sequence ctDNA from over 1000 clinical CSF samples from patients with and without clinically documented CNS involvement by cancer.

## Materials and methods

The vast majority of clinical CSF samples were obtained by lumbar puncture and submitted for routine genomic profiling at the Diagnostic Molecular pathology laboratory to prospectively assess for relevant genomic alterations that could inform the diagnosis, classification or treatment of a suspected solid tumor involving the CNS were identified. For all patients, clinical charts were reviewed, when available. Calendar dates related to sample collection were recorded and the pathology reports were used to input patient diagnosis, when available. For each patient, any evidence of CNS involvement by other ancillary methods was collected. When available, molecular testing from prior samples sequenced in the laboratory were reviewed and correlated at the time of sign out.

All molecular laboratory procedures, volumes of samples received, extraction details, sequencing metrics and results were recorded, and electronic records were maintained in accordance to defined requirements for clinical testing.

This study was approved by the Institutional Review Board at Memorial Sloan Kettering Cancer Center (MSKCC). Written informed consent for the use of genomic data for research (12–245 or 06–107 consents) was obtained for all patients; patients were not compensated for participation.

### CSF collection/delivery/storage

Per protocol, CSF samples were collected in Streck tubes, shipped and stored at room temperature. Samples were processed within 24 h of receipt, when feasible. Samples collected in sterile body fluid containers were occasionally accepted if internally collected and processed upon arrival to the extraction lab or immediately transferred to Streck tubes for subsequent processing.

### cfDNA isolation

CSF was separated from its cellular constituents by double centrifugation (10 min at 1600 g and 10 min at 3000 g at 22 °C) to limit genomic DNA contamination. Cell-free DNA (cfDNA) was manually extracted from the supernatant using MagMax cfDNA Kits (Thermo Fisher Scientific, Waltham, MA), according to manufacturer’s protocol and suspended in water in a 55 μL volume.

### Assessment of cfDNA concentration and size distribution

Fragment analysis of cfDNA was performed using a High Sensitivity D1000 Screen Tape and corresponding reagents, which are loaded onto a 4200 Agilent Tape Station (Agilent, Santa Clara, CA) [10]. A 2 µL aliquot of extracted DNA per sample was analyzed to produce an electropherogram of the size distribution of the DNA fragments. Gating was then applied to assess the proportion of fragments within the cell free range of DNA (100–700 bp) and this compared with the total sample DNA (50–1000 bp) to estimate the percentage of cfDNA.

### Sample sequencing and analysis

Genomic sequencing was performed using the MSK-IMPACT™ solid tumor assay, a custom hybridization capture-based panel targeting all coding regions of 468 (n = 274) or 505 (n = 733)) genes and select intronic regions for detection of point mutations, small insertion and deletions, relevant fusions and structural rearrangements and genome wide copy number changes. Testing using this assay was approved by the New York State Department of Health for clinical sequencing of genomic DNA (gDNA) from formalin fixed paraffin embedded (FFPE) tumor and other gDNA sources, as well as cfDNA isolated from CSF and is performed in our CLIA-compliant Molecular Diagnostics Service laboratory with processes detailed previously [3, 9]. Captured libraries of both cfDNA and gDNA isolated from blood (patient specific control) were sequenced as tumor:normal pairs on Illumina HiSeq 2500 or NovaSeq 6000 instruments and the data was analyzed according to established and published methods [3, 9]. Point mutations and indels were detected using MuTect, Vardict and SomaticIndelDetector. SCNA were detected by comparing loess-normalized sequence coverage of targeted loci of the tumor with a standard diploid non-tumor sample. Structural variants (SV) were identified using DELLY [16]. Variants were annotated using VEP/ ANNOVAR; variants that were considered germline or due to clonal hematopoiesis in the patient-matched blood normal sample were filtered out. All variants were reviewed and called by a bioinformatic analyst and a board-certified molecular genetic pathologist. Tumor mutational burden (TMB) was calculated as mutations per megabase (Mb) utilizing only nonsynonymous coding mutations in the calculations (including frameshift, point mutations, and small insertions and deletions (indels)).

### Determination of clinical actionability

Sequence mutations, copy number alterations, and rearrangements were annotated according to OncoKB, an FDA recognized human genetic variant database with curated content relating to the oncogenic effects and treatment implications of somatic alterations (http://oncokb.org) [7, 20]. Briefly, mutations were annotated based on level of evidence that supports the use of an existing drug either as standard or care (levels 1 or 2), investigational (levels 3A and 3B) or hypothetical (level 4, denoting compelling evidence to support response to a drug). In addition, specific alterations may also be annotated as predictive of resistance to an FDA approved drug (R1) as standard or care or R2 denoting investigational resistance. Specific details may be obtained from the above website.

### Analysis of mutational signatures

Mutational signatures were analyzed as per the protocol utilized in Zehir et al*. *[22]. In brief, we initially determined the tumor mutational burden for each CSF sample and ≥ 13.8 mutations/Mb was considered a sufficiently high TMB for signature analysis. Then, based on the pattern and context of synonymous and non-synonymous mutations across the targeted genome of CSF samples with elevated TMB, we sought to identify the most enriched signature per sample, according to the 30 mutational signatures described previously with a threshold set at 40% [2].

### MSK-ACCESS

cfDNA analysis from plasma was performed on a subset of cases using the MSK-ACCESS assay. This is a duplex barcoded, hybrid-capture sequencing panel, validated for detection of somatic alterations in plasma cfDNA and includes 129 key cancer-associated genes selected from the MSK-IMPACT assay. Details of the assay are further described in prior published work [17].

### Statistical analysis

Overall survival (OS) of patients was defined from the date of CSF collection until the date of death or the date of censoring, which was the most recently recorded visit at MSKCC if death was not recorded in the electronic medical record. For patients with multiple CSF samples, the first CSF sample was selected for analysis. Comparison of OS by variables of interest was performed using the Log-Rank test as well as with Cox proportional hazards regression modeling to estimate hazard ratios (HR) and 95% confidence intervals (CI). We compared the OS of patients with a clinically detectable genetic alteration in the CSF to patients without, followed by a breakdown by tumor type. Heterogeneity of the association of clinically detectable genetic alteration with OS by tumor type was formally tested with an interaction term.

For studying the associations between variables of interest and CSF ctDNA positivity in the lung cancer cohort, we used repeated measures generalized estimating equations with logit link and exchangeable covariance matrix. Univariable GEE models estimated odds ratios (OR) and corresponding 95% confidence intervals (CI). These analyses were performed in SAS v9.4 (Cary, NC). All other analyses, such as Kaplan–Meier survival analyses, Log-rank tests, and other modeling were performed using R v4.1.2 (The R Foundation). All statistical tests were two-sided with an alpha level of statistical significance set at < 0.05.

## Results

### Patients

Between November 2018 and November 2022, we collected 1062 CSF samples from adult and pediatric patients seeking oncology care or molecular pathology consultation at Memorial Sloan Kettering Cancer Center (MSK); 55 CSF samples were excluded from further genomic profiling for logistic or administrative reasons. The final sequenced cohort included 1007 CSF samples from 711 patients (Supplementary Fig. 1a-b). We collected multiple CSF samples in 150/711 (21.1%) across their disease course (median: 2, range: 2–12 samples per patient) (Supplementary Fig. 1c). All CSF samples were processed and sequenced upon receipt in the clinical laboratory, in parallel with the corresponding matched normal (blood) sample.

Our patient cohort included patients with over 90 distinct tumor types of primary CNS and metastatic origin, including lung cancer (n = 188), breast cancer (n = 150) and gliomas (n = 148) as the most common broad categories (Supplementary Table 1). 85/1007 (8.5%) of the CSF samples were collected from patients who did not have any clinically documented evidence of CNS involvement by cancer. The median follow-up following CSF collection was 240 days (IQR: 112–483 days).

### Landscape of somatic genomic alterations detected in CSF

922/1007 (91.5%) CSF samples were collected from patients with clinical CNS disease; of these, 53% (489/922) harbored at least one somatic genetic alteration. These samples were categorized as ctDNA positive (ctDNA +).

By contrast, all samples collected from patients without any clinically documented CNS involvement by cancer (85/85) were negative for genetic alterations (specificity = 100%).

A total of 7110 somatic alterations (3944 somatic non-synonymous mutations, 2980 somatic copy number alterations, and 186 structural rearrangements) were detected across the 489 CSF ctDNA + samples. The number of mutations and variant allele frequencies (VAF) varied across ctDNA + samples, with a median of 4 mutations per sample (IQR: 2–8, range: 1–415) and VAFs ranging from 1 to 100% (median VAF: 38.7%, IQR: 23.2–51.1%). Tumor types varied by rates of ctDNA positivity, with GI cancers having the greatest proportion of CSF samples harboring at least one genetic alteration and embryonal tumors having the fewest. Likewise, VAF varied across the different tumor types with GI cancers having the greatest median VAF compared with other tumor types, possibly reflecting different rates of ctDNA shed across tumor types and the varying propensities for leptomeningeal versus parenchymal-only disease (Supplementary Fig. 2).

**Fig. 1:**
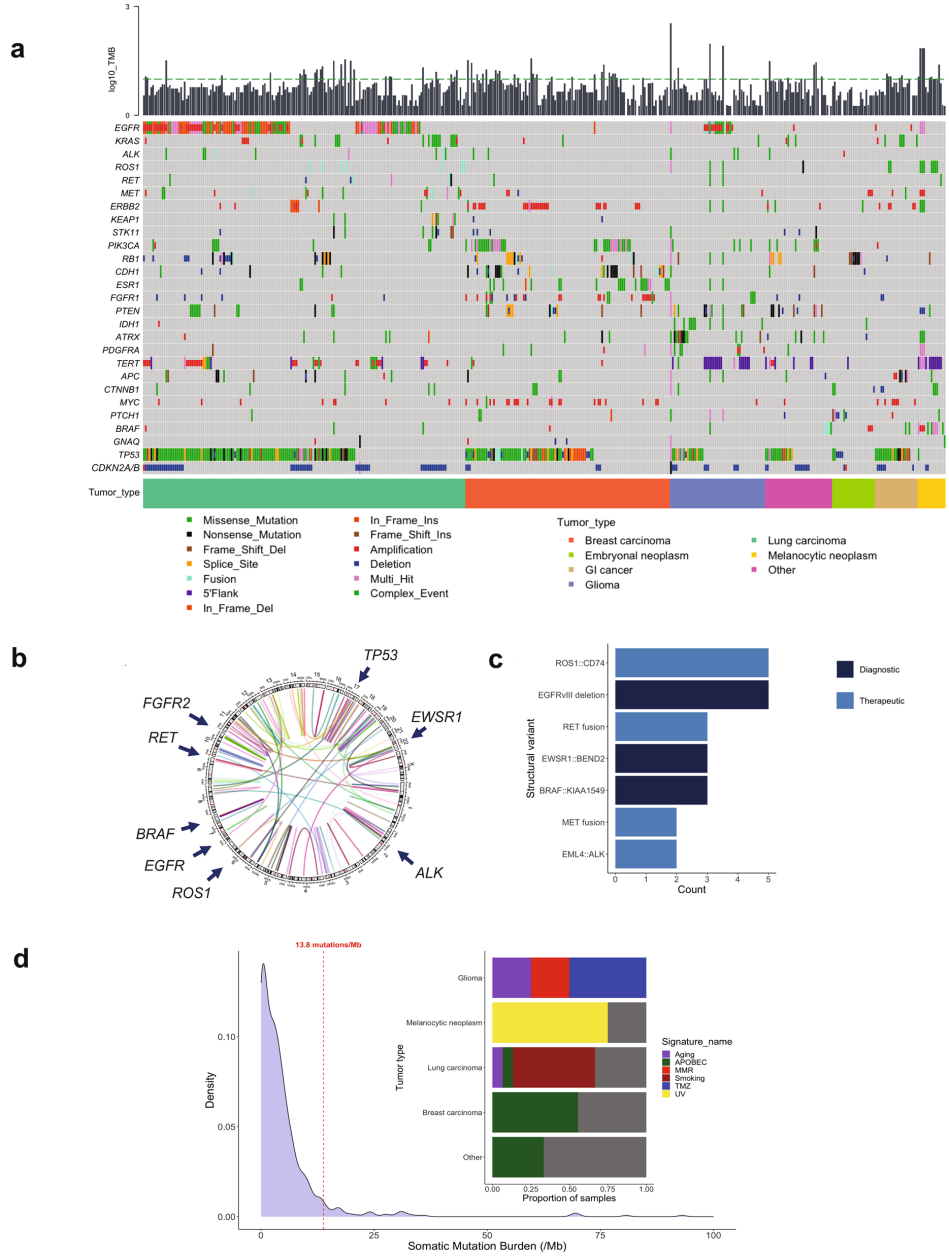
Genomic alterations detected in CSF-ctDNA. **a** Oncoplot of the most frequently altered genes, stratified by broad tumor categories. Each column represents an individual sample. Plot includes non-synonymous mutations, indels, copy number alterations and structural variants. Upper bars depict TMB levels; the dashed green line indicates a TMB of 10 muts/Mb. Lowest track indicates the tumor category. Multi_Hit refers to those genes that were mutated more than once in the same sample. **b**, **c** Circos plot of the 186 structural variants identified. Arrows highlight select recurrent alterations with the most clinical relevance, further stratified by number and clinical implication as diagnostic or therapeutic. **d** Distribution of observed mutation rates across CSF samples sequenced; a threshold of 13.8 mutations/Mb was considered indicative of high mutation burden based on historical analysis of 10,000 tumor samples by MSK-IMPACT testing (left). Dominant mutation signatures identified in cases with high mutation burden. The percent of cases harboring a dominant mutation signature is shown for each broad tumor category (right panel). MMR: Mismatch repair deficiency; UV: Ultraviolet light; TMZ: Temozolomide

We observed the full spectrum of genetic alterations in CSF ctDNA. *TP53* was the most frequently altered gene across all tumor types (n = 242, 49% of 489 ctDNA + samples). Other commonly mutated genes were consistent with the expected landscapes of individual tumor types. For instance, both mutation and high amplification could be detected in *EGFR*, *MET*, and *ERBB2* in lung cancer, as well as mutations in *KRAS*, *BRAF*, *STK11*, *KEAP1* and many others. Mutations in *PIK3CA p.E545K* and amplification in *ERBB2* were common in breast cancers, *BRAF p.V600E* in metastatic melanoma and *IDH1 p.R132H* in IDH-mutant gliomas (Fig. 1a).

Based on assay design and coverage of key intronic regions of the genome, we were also able to detect a broad range of clinically relevant fusions, such as *EML4::ALK*, *RET*, and *ROS1* rearrangements with diverse gene partners in lung carcinomas, *BRAF::KIAA1549* and *EGFRvIII* alterations in gliomas (Fig. 1b and c).

Beyond the individual somatic alterations, we interrogated CSF samples with the highest tumor mutation burden (≥ 13.8 mutations/Mb, n = 35) for the presence of mutational signatures and identified signatures related to prior exposure to ultraviolet (UV, n = 3), APOBEC (n = 7), smoking (n = 8), and temozolomide (n = 2) in metastatic cutaneous melanomas, breast cancers, lung cancers, and gliomas, respectively (Fig. 1d). The threshold of 13.8 mutations/Mb was chosen as indicative of high mutation burden, based on historical analysis of 10,000 tumor samples by MSK-IMPACT testing [22].

### Clinical relevance of CSF-ctDNA positivity

To assess the clinical relevance of somatic variants in CSF, we annotated each of the alterations detected by their level of clinical actionability according to the OncoKB (https://www.oncokb.org/) precision oncology knowledge base [7]. OncoKB was recognized by the Food and Drug Administration as a tumor mutation database that provides information about the biological and clinical implications of over 5,000 cancer gene alterations. Level 1 alterations in OncoKB are defined as FDA-recognized biomarkers predictive of response to an FDA-approved drug in this indication. Level 2 alterations are standard care biomarkers recommended by the NCCN or other professional guidelines predictive of response to an FDA-approved drug in this indication. Level 3A alterations require compelling clinical evidence to support the biomarker as being predictive of response to a drug in this indication.

**Fig. 2:**
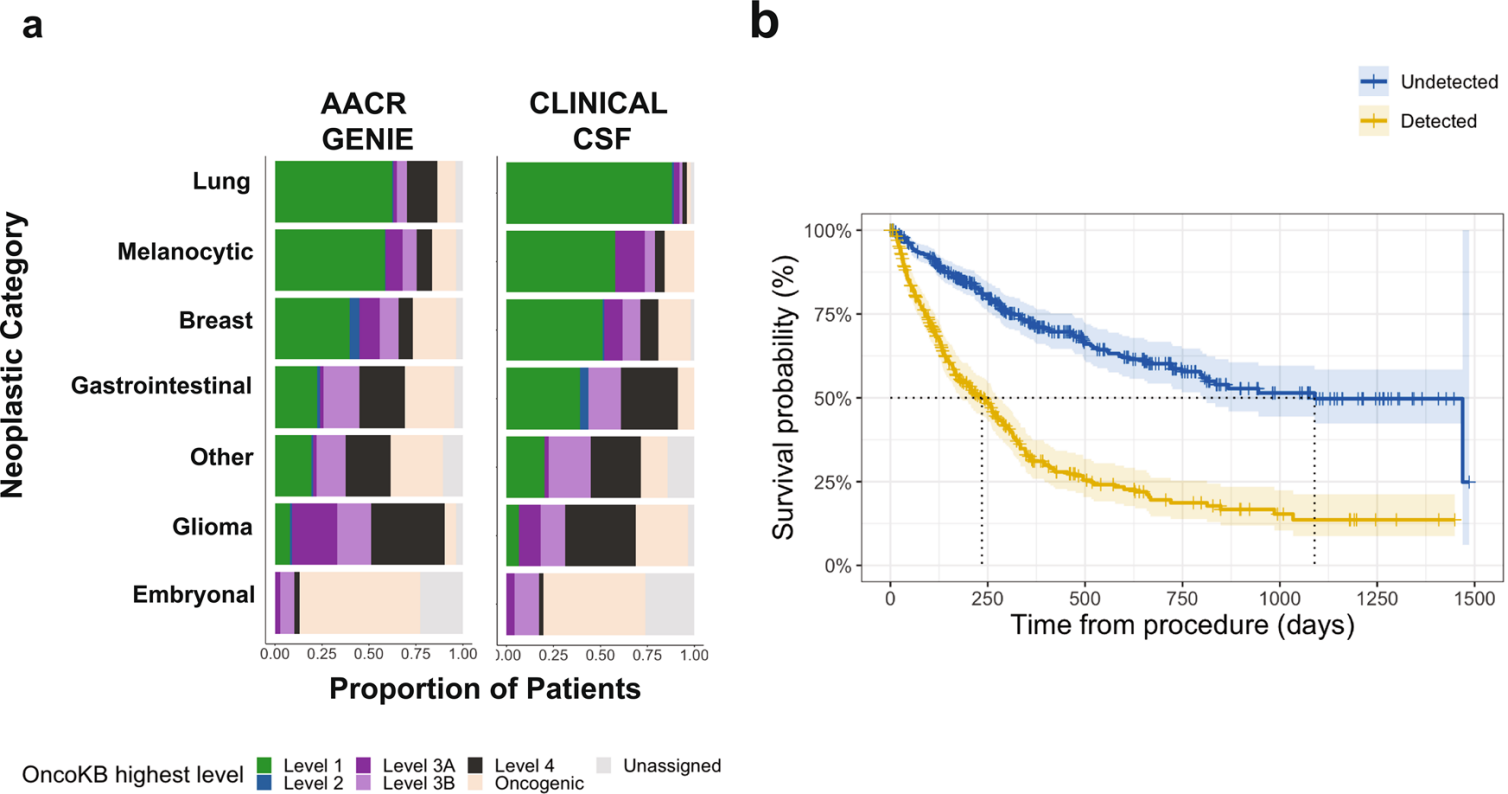
Clinical Relevance of CSF-ctDNA positivity. **a** Alterations detected in cfDNA from CSF were annotated and stratified by their level of clinical actionability according to the OncoKB precision oncology knowledge base. The proportions were compared to the AACR-Genie MSK cohort of solid tumor (n = 47,271). There is a relative enrichment for level 1 alterations due to the use of the assay for monitoring of patients on targeted therapies. **b** Survival curves showing that detection of ctDNA in CSF is associated with lower survival probability

Across the 489 ctDNA + samples, 248 (50.7%) had a level 1 OncoKB actionable alteration. Lung carcinomas had the highest level 1 actionability in the cohort, consistent with the number of precision oncology drugs currently available in lung cancer. In this subset, we also detected the highest number of alterations predictive of therapeutic resistance (OncoKB R1 and R2) which informed further patient management. Among other malignancies, the OncoKB levels of actionability in the CSF were broadly comparable by cancer type to those found in tissues sequenced by MSK-IMPACT as part of the AACR GENIE cohort (n = 47,271) (Fig. 2a).

In addition to the assessment of therapeutic actionability, genomic profiles provided pivotal information for tumor subclassifications. For both primary and suspected metastatic lesions, profiling established clonal relatedness to a known malignancy and, in some cases, informed the presence of a previously unsuspected tumor (Supplementary Figs. 3 and 4).

Notably, given the relative purity of ctDNA and the high number of genetic alterations that could be detected in some cases, the assessment of mutational signatures assisted in the determination or confirmation of the primary tumor site, such as UV signatures or smoking signatures in suspected metastatic cutaneous melanoma or lung adenocarcinoma, respectively (Supplementary Fig. 5).

We determined the relationship between ctDNA positivity and overall survival (OS) in our cohort. Across all cancer types, detection of a genetic alteration in the CSF was associated with a three-fold increased risk of death (HR: 3.23, 95% CI: 2.58–4.05, *P* < 0.001). Median survival was 854 days shorter in patients with CSF positivity than otherwise (detected alteration: 235 days (95%CI: 177–272 days); undetected alteration: 1089 days (95%CI: 796 days-not reached) (Fig. 2b) (Supplementary Table 2). The association between shortened OS and CSF positivity was seen across all tumor subtypes (except embryonal and GI cancers) with no statistically significant heterogeneity (*P* = 0.13, Supplementary Fig. 6).

### CSF sampling in patients with metastatic lung cancer

Patients with lung cancer represented the largest subgroup of patients in our dataset and the subgroup of patients with the most FDA-approved genotype-directed therapies. This allowed a closer look into the role of CSF sampling as a tool for CNS assessment and disease monitoring.

Among the lung cancer patients those with parenchymal brain metastases and additional evidence of leptomeningeal involvement (defined by positive cytology, positive circulating tumor cells ≥ 3, or radiographic leptomeningeal spread as called by the formal clinical radiology report) were more likely to have positive CSF ctDNA than those with parenchymal brain metastases in the absence of leptomeningeal involvement (OR: 20.17; CI: 9.65–42.16; *p* < 0.0001) (Supplementary Table 3, Additional File 1). After excluding cytology samples that were reported as atypical or suspicious by pathology (n = 33), detection of a genetic alteration in the CSF had greater sensitivity than positive cytology for the presence of leptomeningeal disease (sensitivity: 85.4% vs. 61.7%) and greater negative predictive value (80% vs. 66%). Since ctDNA positivity also occurred in some patients with parenchymal-only disease, this finding was not entirely specific for leptomeningeal disease (specificity: 78.7%, positive predictive value: 84.4%).

**Fig. 3:**
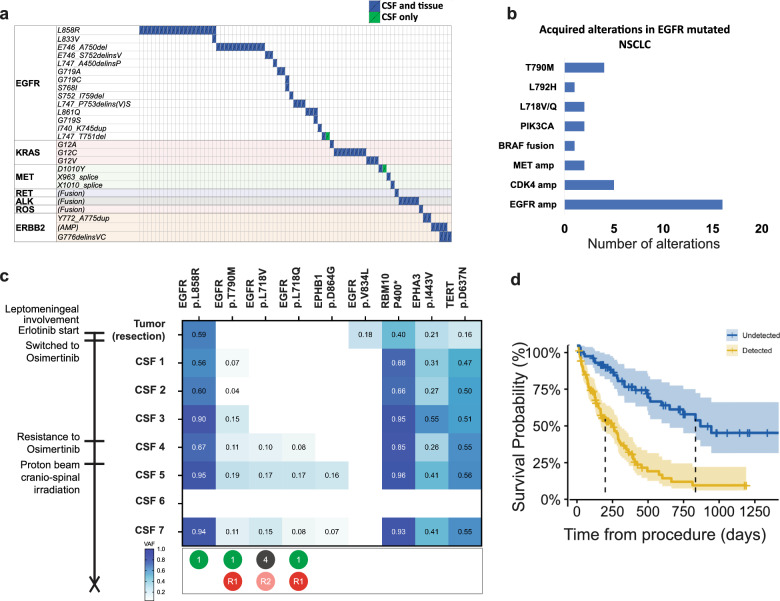
CSF sampling in NSCLC. **a** 77 samples with actionable driver alterations detected in CSF were compared to results from prior tissue biopsies. Driver alterations initially detected in the tumor tissue were universally detected in the CSF. Each column represents 1 patient. Blue boxes designate those samples where both CSF and tissue sequencing demonstrated the same driver alteration. In two cases, the mutation detected in the CSF was distinct form the one detected in the tumor. In both cases, retrospective review demonstrated the presence of multifocal lung disease with the metastasis representing a separate primary that was not previously sequenced. **b** Among patients with EGFR sensitizing mutations, sequencing of CSF from 28 patients detected several additional alterations associated with secondary resistance, including mutations in EGFR, and alterations in other genes *(MET, PIK3CA, BRAF)*. **c** Representative case of a patient with EGFR mutated lung adenocarcinoma and monitoring starting at the time of suspected CNS metastasis. 7 CSF samples are obtained demonstrating the gradual emergence of several resistance mechanisms associated with treatment with EGFR inhibitors (T790M, L718V and L718Q. The table displays the mutations detected in each sample sequenced, along with the corresponding VAF’s (%), highlighted according to the color scale (bottom left). Lowest track denotes the classification of the EGFR mutations as sensitizing (L1, green) or associated with acquired resistance (R1 standard care resistance; R2 investigational resistance, red) according to OncoKB. L4 (dark gray) denotes an alteration with compelling biological evidence that supports the biomarker as being predictive of response to a drug. **d** Survival curves for NSCLC patients demonstrate that detection of ctDNA in CSF is associated with lower survival probability

In lung cancer patients, the driver alterations initially detected in the tumor tissue were universally detected in the CSF (Fig. 3a). Among lung cancer patients with EGFR sensitizing mutations and on therapy, CSF sequencing demonstrated the emergence of gatekeeper mutations associated with acquired resistance, including *EGFR p.T790M*, *p.C797S*, *p.L792H*, *p.L718Q*, *p.L718V* and p.*G724S*. Other acquired alterations included amplifications in *MET, and EGFR*, and off target alterations in *BRAF* (fusion), *KRAS, PIK3CA* and others (Fig. 3b). Some patients harbored several alterations as exemplified by a patient with EGFR-mutant non-small cell lung carcinoma where repeated CSF sequencing identified the emergence of multiple different EGFR mutations in response to first and third-generation EGFR inhibitors (Fig. 3c). Among patients with ALK fusions and *MET* exon 14 skipping mutations, emergence of additional ALK mutations (*p.G1202R* and *p.G1269A*) and *MET* alterations (*p.Y1230N)* were also detected upon progression on targeted therapy (Supplementary Fig. 7).

NSCLC patients with positive CSF-ctDNA had significantly shorter survival following CSF collection than NSCLC patients with negative CSF-ctDNA (Fig. 3d).

### Comparison of CSF ctDNA with plasma and tumor tissue

Several patients in our cohort had undergone a tumor biopsy or plasma collection within 90 days of the CSF collection. This provided an opportunity to compare the representation of the cancer genome in CSF compared to tumor tissue or blood.

Correlations between somatic alterations in CSF ctDNA and tumor DNA were possible for 56 pairs (55 patients). Overall, we detected 999 alterations in tumor and CSF from these patients. 434/999 (43%) alterations were shared between tumor and CSF, 273/999 (27%) were private to the CSF and 292/999 (29%) were private to the tumor biopsy (Supplementary Fig. 8a). The frequency of shared CSF/tissue alterations was considerably higher (41/53 = 77%) than private alterations to CSF or tissue for the most clinically relevant alterations (OncoKB levels 1 to 3A) (Supplementary Fig. 8b). A comparison of VAFs for shared mutations revealed significantly higher levels in ctDNA from CSF (median VAF: 32%, IQR: 27%) compared to the tumor tissue (median VAF: 24%, IQR: 33%), despite routine enrichment by manual macro-dissection in solid tumor samples where necessary (*P* < 0.01, Mann–Whitney U test) (Supplementary Fig. 8c-d). Measurements of tumor mutation burden (TMB) in tumor tissue and CSF corresponded closely with each other (r = 0.81, *P* < 0.001, Spearman’s rank correlation) (Supplementary Fig. 8e.

Comparisons of somatic alterations detected in ctDNA from CSF *versus* plasma was performed on 31 patients and focused on the somatic mutations targeted by both assays (MSK-IMPACT and MSK-ACCESS). Over half of the total alterations were shared between plasma and CSF (77/142, 54%) (Supplementary Fig. 9a), and included the majority of clinically relevant mutations (24/32 mutations (75%), OncoKB levels 1 to 3A) (Supplementary Fig. 9b). Compared to the alterations identified in plasma, mutations detected in CSF cfDNA were identified at significantly higher VAFs (CSF: median VAF = 36.4%, IQR = 34.3% vs. Plasma: median VAF = 2.3%, IQR = 10.7%, *P* < 0.01, Mann–Whitney U test), likely reflecting greater dilution of tumor-derived DNA by non-neoplastic DNA in blood (Supplementary Fig. 9c).

### Determinants of CSF-ctDNA positivity

**Fig. 4:**
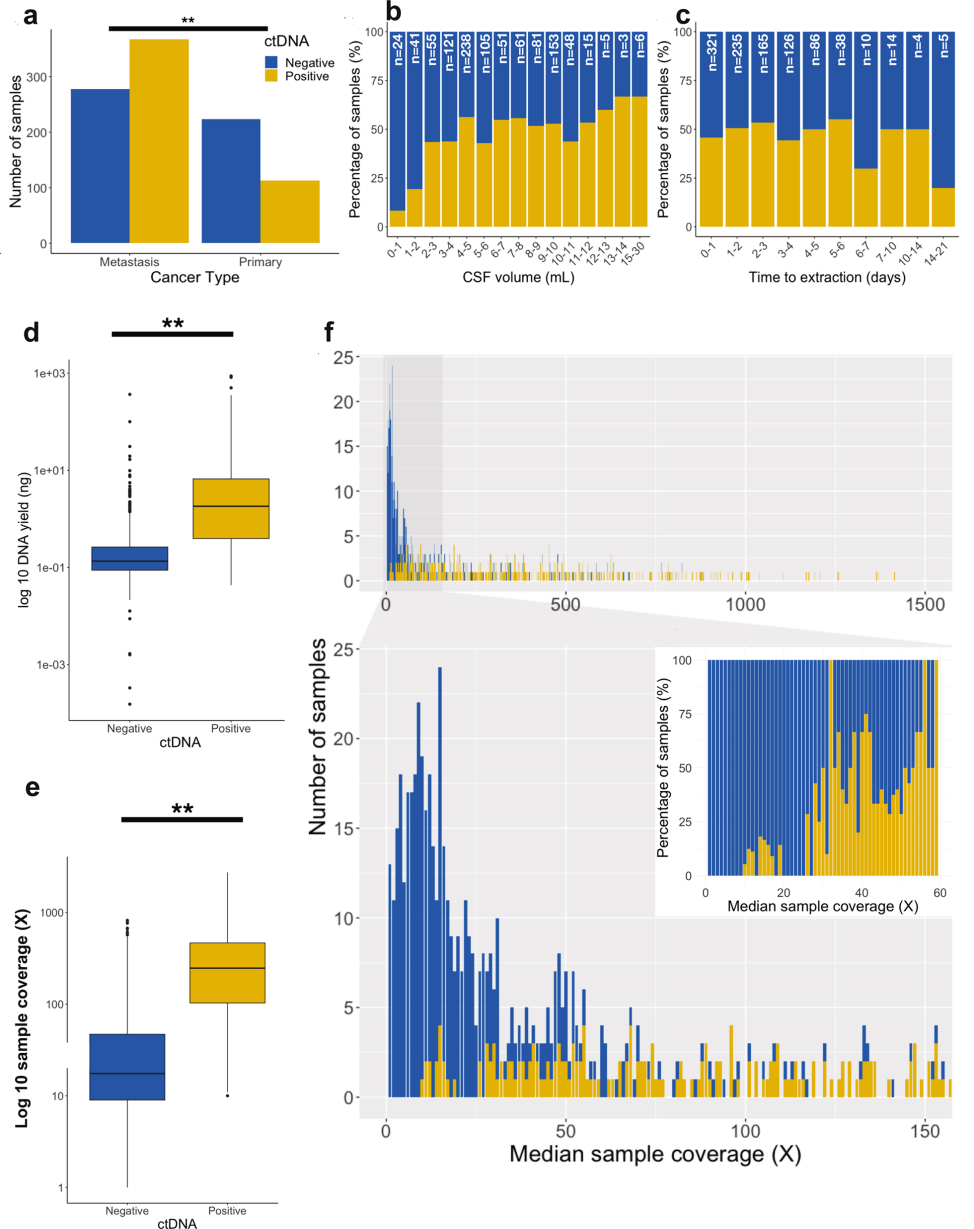
Pre-analytic factors associated with CSF-ctDNA positivity. **a** Stratification of samples based on disease type (primary CNS tumor vs metastasis) shows that that metastatic tumors have higher rates of ctDNA positivity than primary tumors. **b** Samples are stratified by the volume of CSF received for testing. While genetic alterations could be detected even in the context of very low volume samples, the rate of positivity was critically impacted for those samples below 2 ml. These samples were associated with rates of ctDNA positivity between 8 and 20%. The rate of positivity increases as volumes reach 5 ml and above. Yellow bars indicate the proportion of samples that are ctDNA positive. Blue bars indicate those that are ctDNA negative (no detected genetic alterations). **c** Analysis of rates of positivity for samples according to time to extraction. Across the entire cohort, increased time to extraction was not associated with increased proportion of ctDNA negative samples. Samples extracted outside the stability criteria of STREK tubes (> 14 days) constituted a very small proportion of the samples–this very small subset demonstrated a drop in the rate of positivity compared to those extracted before 14 days but the number was too low for a conclusive analysis. **d**, **e** Comparisons of total DNA yields and sequencing coverages between ctDNA + and ctDNA- samples. Overall, the proportion of ctDNA + samples increased with higher DNA yield and, consequently, higher sample coverages. **f** A broad range of coverages are found across CSF samples. Top graph shows the range of coverages across the entire cohort. Lower panel and insert (right) display the zoomed views of the samples with lowest coverages. Despite the low coverages, detection of genetic alterations remains possible in many cases below 50× due to the high proportion of ctDNA in CSF samples (not diluted by cfDNA from hematopoietic components). Higher proportion of samples have ctDNA detected when sample coverage increases

The rates of CSF ctDNA positivity among patients with primary CNS tumors versus CNS metastasis were compared, excluding the small subset of cases with tumors of unknown primary origin (n = 26). Samples from patients with CNS metastasis were more likely to be ctDNA positive (OR = 2.60, CI 1.96–3.46, *P* < 0.001, Fisher’s exact test) (Fig. 4a).

While our standard recommendation for sample collection was 10 mL of CSF in Streck tubes, we received highly variable sample volumes (Fig. 4b). The median CSF volume submitted was 5.5 mL (range 0.4–30 mL, IQR: 4.5 mL). Most samples (99.0%) were received in Streck BCT tubes; 1.0% arrived in sterile containers and were immediately transferred to Streck tubes in our lab; 99.2% of samples were processed within the established stability window of the Streck collection tubes (≤ 14 days). Delays in processing reflected lags in shipping and transport when samples were procured at outside hospitals; 45 samples (4.5%) were flagged due to deviations in quality control (received in non-Streck tubes, blood-tinged samples or frozen Streck tube collections).

In this cohort, delays in CSF DNA extraction did not adversely affect the rate of CSF ctDNA positivity (r = 0.27, *P* = 0.29, Spearman’s rank correlation) (Fig. 4c). However, the proportion of ctDNA in the samples decreased with increasing time to extraction (r = − 0.07, *P* = 0.03, Spearman’s rank correlation) beyond 7 days, likely related to dilution associated with gDNA released from cells when CSF is not separated. For the small subset of samples submitted in non-Streck tubes and those visually bloody, we observed significantly lower VAF’s compared to Streck collections (median VAF = 23.5% vs. 38.9%, respectively, *P* < 0.001, Mann–Whitney U test).

While genetic alterations could be detected even in the context of very low volume samples, the rate of positivity was critically impacted for those samples below 2 ml. For these low volumes, samples from patients with CNS disease that were appropriately collected in Streck tubes were associated with rates of ctDNA positivity as low as 8.3% and 20.0% for primary and metastatic CNS tumors, respectively. The rate of positivity increased to 37.8% and 68.6% for primary and metastatic tumors, respectively, as volumes increased to 5 ml. We noticed no significant improvements in ctDNA positivity rates with volumes of 10 ml and above.

The median cfDNA yield was 0.29 ng (range: 0 to 872.5 ng, IQR: 0.11–2.19 ng). All samples were sequenced regardless of the DNA quantity recovered. The median sequencing coverage was 65X (range: 0- 2735X; IQR: 16-295X); 457 samples had coverages below 50X, corresponding to those with lowest cfDNA yields (median DNA yield 0.12 ng [IQR: 0.08–0.19 ng] vs. 1.69 ng [IQR: 0.45–6.03 ng] in samples with greater than 50X coverage).

Comparisons of DNA yields, and coverages between ctDNA + and ctDNA- samples are summarized in Fig. 4d–f and Supplementary Table 4. Overall, the proportion of ctDNA + samples increased with higher sample coverages, while sample coverage correlated strongly with DNA yield (r = 0.79, *P* < 0.001, Spearman’s rank correlation).

## Discussion

Our study reports on the clinical implementation of prospective CSF ctDNA sequencing for the assessment of CNS tumors. We identified genetic alterations in 53% of samples with known CNS involvement by cancer. In contrast, we did not detect CSF ctDNA in any of the patients without clinically documented evidence for CNS involvement by cancer. Our examination of over 1,000 CSF samples identified several scenarios where CSF ctDNA detection provides clinically actionable information. In CSF ctDNA-positive cases, the distribution of somatic alterations was consistent with the full spectrum of tumor-type specific alterations. Sequencing of CSF ctDNA identified genetic alterations associated with acquired resistance to molecularly targeted therapy, provided a reliable measure of tumor mutation burden (TMB), and identified mutations signatures associated with prior carcinogen exposure.

Patients with lung cancer represented the largest disease subgroup in our study. In this cohort of patients, we observed that patients with metastatic disease and leptomeningeal involvement were more likely to have positive CSF ctDNA than those with parenchymal brain metastases in the absence of leptomeningeal disease. We also observed that detection of ctDNA in CSF was associated with significantly shorter overall survival, suggesting that the detection of CSF ctDNA may be useful as supportive evidence of active CNS disease.

Analyses of contemporaneously collected CSF and plasma samples for a subset of patients in our study suggests that CSF “liquid biopsies” are more suitable for the monitoring of CNS cancer involvement than patient plasma. This conclusion is consistent with prior studies which have shown that plasma ctDNA testing has limited value in the context of primary brain tumors or metastatic lesions confined to the CNS [5]. Surprisingly, among the ctDNA positive CSF samples that were compared with contemporaneously collected tumor tissue, the VAFs across shared mutations was significantly higher in CSF, despite common practices of tumor enrichment of tissue samples by manual microdissection prior to sequencing. These findings highlight some of the unique qualities of CSF for genomic applications. CSF is a largely acellular fluid with virtually absent cell free DNA contributions from hematopoietic cells (compared to plasma) and very low DNA component from normal CNS cells. As such, in the context of CNS involvement with tumors shedding DNA into the CSF space, the DNA is tumor enriched and therefore genetic alterations are observed at very high variant allele frequencies. By contrast, tumor cells in tissue are frequently associated with other cells within the invaded organ and not amenable to enrichment by manual microdissection techniques.

We have identified pre-analytical factors that affected CSF ctDNA positivity, including the amount of CSF volume submitted for testing, ctDNA recovery and DNA sequencing coverage. While delays in CSF processing and improper collections did not necessarily affect our rate of positivity, they were associated with lower ctDNA component, which would impact the overall sensitivity of the assays. While these associations are not surprising, they raise important considerations for optimizing CSF collections. The use of more targeted assays with higher sensitivity may also be valuable, although sensitivity remains contingent on recovering sufficient quantity of cell free DNA for testing. For example, the use of digital PCR assays on pre-capture NGS libraries could be used to confirm key subthreshold variants that do not meet calling criteria with our established analysis pipelines.

In summary, our study provides a foundation for further improvements of CSF ctDNA assays and illustrates the feasibility and potential utility of incorporating CSF ctDNA profiling into the evaluation of patients with CNS cancer.

## Supplementary Information

Supplementary Material 1: Supplementary Figure 1. Study Overview.Summary of the MSK-IMPACT™ workflow for tumor matched normal sequencing of ctDNA from CSF. cfDNA from CSF and genomic DNA from normal blood are extracted and sequenced as Tumor:Normal pairs to facilitate the analysis of somatic variants including mutations, somatic copy number alterations and structural rearrangements.CONSORT diagram showing samples excluded from pre-analytic and genomic/clinical analyses in this study.Distribution of samples per patient across the overall cohort

Supplementary Material 2: Supplementary Figure 2. Variability of ctDNA-positivity and VAF across tumor types.Proportion of samples that are ctDNA+ by tumor type.Boxplots showing the distribution of VAFs across tumor types. Boxplots display the median, quartiles and range of values by tumor type

Supplementary Material 3: Supplementary Figure 3. Tumor subclassification using CSF-ctDNA. A 31-year-old woman presented with an expansile T11-T12 intramedullary cord lesion. Biopsy was submitted for sequencing, but the tissue was insufficient for molecular analysis. CSF-ctDNA identified a histone mutation H3-3A p.K28M which, together with the clinical presentation, supported the diagnosis of diffuse midline gliomaaccording to the 2021 WHO Classification of Tumors of the Central Nervous System. Sequential monitoring by serial CSF sampling demonstrated increasing mutational load and VAF with disease progression. Throughout this time, cytologic assessment of the CSF remained negative

Supplementary Material 4: Supplementary Figure 4. Diagnosis of primary tumor using CSF-ctDNA. This 44-year-old man presented to the hospital with a diagnosis of metastatic papillary thyroid carcinoma. Sequencing of genomic DNA from the tumor demonstrated a canonical BRAF p.V600E mutation. In subsequent months, the patient presents with a brain lesion suspected to represent a brain metastasis. CSF sequencing revealed a mutational profile that was distinct from and clonally unrelated to the papillary thyroid cancer, with a non-canonical histone mutation; these variants and the lack of the BRAF mutation indicated a second malignancyfor this patient

Supplementary Material 5: Supplementary Figure 5. Determining clonal relatedness of CNS metastasis to a primary tumor using CSF-ctDNA. A 58-year-old man with history of thyroid carcinoma presented a metastatic bone lesion. Histological and immunohistochemical evaluation of the biopsy was non-conclusive, favoring an adenocarcinoma. Plasma testing was initiated and detected 4 mutations with non-specific profile. Sequencing of the sacral lesion did not reveal a driver alteration but pointed toward a metastasis from a lung primary site based on a weak smoking mutational signature. A few months later, the patient developed symptoms concerning for leptomeningeal involvement. Cytology showed rare, atypical cells; CSF-ctDNA identified 28 genetic alterations with high overlap to the sacral tumor profile and with a strong smoking-related mutational signature establishing clonal relatedness between. Together, this data established the diagnosis of metastatic NSCLC to the CNS.Outlines the sequence of events.Details of the sequencing results in order of availability. Note the marked difference in variant allele frequenciesidentified in the CSFcompared to the sacral biopsy. The table displays the mutations detected in each sample sequenced, along with the corresponding VAF’s, highlighted according to the color scale.Proportion of the genetic alterations identified in the tumor stratified by the type that would support independent mutational signatures. The estimated tumor mutation burdenfor this sample was 18.1 mutations per megabase. 65% of the mutations constituted C>A, G>T, CC>AA, and GG>TT transversions, supporting smoking-induced damage mutagenesis

Supplementary Material 6: Supplementary Figure 6. Detection of ctDNA shortens OS, irrespective of tumor type

Supplementary Material 7: Supplementary Figure 7. Monitoring of Drug Resistance using CSF-ctDNA. Shown is a representative case of a patient with lung adenocarcinoma with Met exon 14 skipping and concurrent MET amplification.Summary of the clinical course.sequencing results for the diagnostic tumor sample, liver metastasis and 4 CSF samples tested during the monitoring phase. Sequencing of the second CSF sample detected the emergence of a new MET alteration Y1230N while on crizotinib. The development of the MET mutations was only detected on the CSF but not in the metastatic lesion from liver. Given the documented leptomeningeal progression, the patient was transitioned to capmatinib.Demonstrates the copy number plot obtained from a CSF sample. Given the high ctDNA in CSF samples, high gains and deep losses can be readily observed. RT1 Palliative radiation to left femur. RT2 Palliative radiation therapy to metastatic cerebellar lesion, SRS3 Stereotactic radiosurgery to new metastatic cerebellar lesion and right trigeminal leptomeningeal lesion

Supplementary Material 8: Supplementary Figure 8. Comparison between Tumor DNA and CSF ctDNA. The data represents 56 tumor/CSF pairs from 55 patients who underwent collection of both samples within 90 days.Venn diagram showing the overlap of mutations between CSF and tumor. Overall, a total of 999 alterations were detected, 434/999alterations were shared between tumor and CSF, 273/999were private to the CSF and 292/999were private to the tumor biopsy.alterations are stratified based on level of actionability. The number of total mutations denoted on the x axis. When considering only those alterations with OncoKB levels of 1 to 3A, the frequency of shared CSF/tissue alterations was considerably higherthan private alterations to CSF or tissue.Comparison of mutational VAFs and their associated OncoKB levels between paired tissue and CSF samples.Comparison of VAFs for shared mutations reveals significantly higher levels in ctDNA from CSFcompared to the tumor tissue, despite routine enrichment by manual macro-dissection in solid tumor samples where necessary.Measurements of tumor mutation burdenin tumor tissueand CSFcorresponded closely with each other

Supplementary Figure 9. Comparison between Plasma ctDNA and CSF ctDNA. The data represents plasma ctDNA/CSF-ctDNA pairs from 31 patients/40 samples, breast carcinoma, GI cancerand the remaining 2 patients having CNS embryonal tumors) who underwent collection of both samples within 90 days. This analysis is restricted to the genomic regions covered by both assays.Venn diagram demonstrates that over half of the total alterations detected were shared between plasma and CSF.Alterations are stratified by level of actionability. All level 1 alterations are either shared or are private to the CSF.Comparison of VAFs for shared mutations reveals significantly higher levels in ctDNA from CSF Compared to the alterations identified in plasma.

Supplementary Material 10. Supplementary tables.

Supplementary Material 11. Additional file 1: Lung cohort with clinical and cytology data.

Supplementary Material 12. Additional file 2: Glioma cohort with tissue and CSF disease-relevant genomic comparisons.

## Data Availability

Somatic alteration data (including mutations and allele specific copy number calls) will be uploaded to cBioPortal for Cancer Genomics. Raw sequencing data cannot be broadly available due to privacy laws; patient consent to deposit raw sequencing data was not obtained.

## References

[1] Aizer AA, Lamba N, Ahluwalia MS, Aldape K, Boire A, Brastianos PK, Brown PD, Camidge DR, Chiang VL, Davies MA et al (2022) Brain metastases: a society for neuro-oncology (SNO) consensus review on current management and future directions. Neuro Oncol 24:1613–1646. 10.1093/neuonc/noac118 35762249 10.1093/neuonc/noac118 PMC9527527

[2] Alexandrov LB, Nik-Zainal S, Wedge DC, Aparicio SA, Behjati S, Biankin AV, Bignell GR, Bolli N, Borg A, Børresen-Dale AL et al (2013) Signatures of mutational processes in human cancer. Nature 500:415–421. 10.1038/nature12477 23945592 10.1038/nature12477 PMC3776390

[3] Bale TA, Yang S-R, Solomon JP, Nafa K, Middha S, Casanova J, Sadowska J, Skakodub A, Ahmad H, Helena AY (2021) Clinical experience of cerebrospinal fluid-based liquid biopsy demonstrates superiority of cell-free DNA over cell pellet genomic DNA for molecular profiling. J Mol Diagn 23:742–752 33781965 10.1016/j.jmoldx.2021.03.001 PMC8207471

[4] Bale TA, Yang SR, Solomon JP, Nafa K, Middha S, Casanova J, Sadowska J, Skakodub A, Ahmad H, Yu HA et al (2021) Clinical experience of cerebrospinal fluid-based liquid biopsy demonstrates superiority of cell-free DNA over cell pellet genomic DNA for molecular profiling. J. Mol. Diagn. JMD 23:742–752. 10.1016/j.jmoldx.2021.03.001 33781965 10.1016/j.jmoldx.2021.03.001 PMC8207471

[5] Bettegowda C, Sausen M, Leary RJ, Kinde I, Wang Y, Agrawal N, Bartlett BR, Wang H, Luber B, Alani RM et al (2014) Detection of circulating tumor DNA in early- and late-stage human malignancies. Sci Transl Med 6:2224ra224. 10.1126/scitranslmed.3007094 10.1126/scitranslmed.3007094 PMC4017867 24553385

[6] Boire A, Brandsma D, Brastianos PK, Le Rhun E, Ahluwalia M, Junck L, Glantz M, Groves MD, Lee EQ, Lin N et al (2019) Liquid biopsy in central nervous system metastases: a RANO review and proposals for clinical applications. Neuro Oncol 21:571–584. 10.1093/neuonc/noz012 30668804 10.1093/neuonc/noz012 PMC6502489

[7] Chakravarty D, Gao J, Phillips S, Kundra R, Zhang H, Wang J, Rudolph JE, Yaeger R, Soumerai T, Nissan MH et al (2017) OncoKB: a precision oncology knowledge base. JCO Precis Oncol. 10.1200/po.17.00011 28890946 10.1200/PO.17.00011 PMC5586540

[8] Chakravarty D, Solit DB (2021) Clinical cancer genomic profiling. Nat Rev Genet 22:483–501. 10.1038/s41576-021-00338-8 33762738 10.1038/s41576-021-00338-8

[9] Cheng DT, Mitchell TN, Zehir A, Shah RH, Benayed R, Syed A, Chandramohan R, Liu ZY, Won HH, Scott SN et al (2015) Memorial sloan kettering-integrated mutation profiling of actionable cancer targets (MSK-IMPACT): a hybridization capture-based next-generation sequencing clinical assay for solid tumor molecular oncology. J Mol Diagn 17:251–264. 10.1016/j.jmoldx.2014.12.006 25801821 10.1016/j.jmoldx.2014.12.006 PMC5808190

[10] Liu APY, Smith KS, Kumar R, Robinson GW, Northcott PA (2022) Low-coverage whole-genome sequencing of cerebrospinal-fluid-derived cell-free DNA in brain tumor patients. STAR Protocols 3:101292. 10.1016/j.xpro.2022.101292 35463474 10.1016/j.xpro.2022.101292 PMC9026582

[11] Liu R, Rizzo S, Waliany S, Garmhausen MR, Pal N, Huang Z, Chaudhary N, Wang L, Harbron C, Neal J et al (2022) Systematic pan-cancer analysis of mutation–treatment interactions using large real-world clinicogenomics data. Nat Med 28:1656–1661. 10.1038/s41591-022-01873-5 35773542 10.1038/s41591-022-01873-5

[12] Louis DN, Perry A, Wesseling P, Brat DJ, Cree IA, Figarella-Branger D, Hawkins C, Ng HK, Pfister SM, Reifenberger G et al (2021) The 2021 WHO classification of tumors of the central nervous system: a summary. Neuro Oncol 23:1231–1251. 10.1093/neuonc/noab106 34185076 10.1093/neuonc/noab106 PMC8328013

[13] Miller AM, Shah RH, Pentsova EI, Pourmaleki M, Briggs S, Distefano N, Zheng Y, Skakodub A, Mehta SA, Campos C et al (2019) Tracking tumour evolution in glioma through liquid biopsies of cerebrospinal fluid. Nature 565:654–658. 10.1038/s41586-019-0882-3 30675060 10.1038/s41586-019-0882-3 PMC6457907

[14] O’Dwyer PJ, Gray RJ, Flaherty KT, Chen AP, Li S, Wang V, McShane LM, Patton DR, Tricoli JV, Williams PM et al (2023) The NCI-MATCH trial: lessons for precision oncology. Nat Med 29:1349–1357. 10.1038/s41591-023-02379-4 37322121 10.1038/s41591-023-02379-4 PMC10612141

[15] Pentsova EI, Shah RH, Tang J, Boire A, You D, Briggs S, Omuro A, Lin X, Fleisher M, Grommes C et al (2016) Evaluating cancer of the central nervous system through next-generation sequencing of cerebrospinal fluid. J Clin Oncol 34:2404–2415. 10.1200/JCO.2016.66.6487 27161972 10.1200/JCO.2016.66.6487 PMC4981784

[16] Rausch T, Zichner T, Schlattl A, Stütz AM, Benes V, Korbel JO (2012) DELLY: structural variant discovery by integrated paired-end and split-read analysis. Bioinformatics 28:i333–i339. 10.1093/bioinformatics/bts378 22962449 10.1093/bioinformatics/bts378 PMC3436805

[17] Rose Brannon A, Jayakumaran G, Diosdado M, Patel J, Razumova A, Hu Y, Meng F, Haque M, Sadowska J, Murphy BJ et al (2021) Enhanced specificity of clinical high-sensitivity tumor mutation profiling in cell-free DNA via paired normal sequencing using MSK-ACCESS. Nat Commun 12:3770. 10.1038/s41467-021-24109-5 34145282 10.1038/s41467-021-24109-5 PMC8213710

[18] Schaff LR, Mellinghoff IK (2023) Glioblastoma and other primary brain malignancies in adults: a review. JAMA 329:574–587. 10.1001/jama.2023.0023 36809318 10.1001/jama.2023.0023 PMC11445779

[19] Soffietti R, Bettegowda C, Mellinghoff IK, Warren KE, Ahluwalia MS, De Groot JF, Galanis E, Gilbert MR, Jaeckle KA, Le Rhun E et al (2022) Liquid biopsy in gliomas: a RANO review and proposals for clinical applications. Neuro Oncol 24:855–871. 10.1093/neuonc/noac004 34999836 10.1093/neuonc/noac004 PMC9159432

[20] Suehnholz SP, Nissan MH, Zhang H, Kundra R, Nandakumar S, Lu C, Carrero S, Dhaneshwar A, Fernandez N, Xu BW et al (2024) Quantifying the expanding landscape of clinical actionability for patients with cancer. Cancer Discov 14:49–65. 10.1158/2159-8290.Cd-23-0467 37849038 10.1158/2159-8290.CD-23-0467 PMC10784742

[21] Wang Y, Springer S, Zhang M, McMahon KW, Kinde I, Dobbyn L, Ptak J, Brem H, Chaichana K, Gallia GL et al (2015) Detection of tumor-derived DNA in cerebrospinal fluid of patients with primary tumors of the brain and spinal cord. Proc Natl Acad Sci USA 112:9704–9709. 10.1073/pnas.1511694112 26195750 10.1073/pnas.1511694112 PMC4534284

[22] Zehir A, Benayed R, Shah RH, Syed A, Middha S, Kim HR, Srinivasan P, Gao J, Chakravarty D, Devlin SM et al (2017) Mutational landscape of metastatic cancer revealed from prospective clinical sequencing of 10,000 patients. Nat Med 23:703–713. 10.1038/nm.4333 28481359 10.1038/nm.4333 PMC5461196

