# Supplementary figures and images for: Real-world experience with circulating tumor DNA in cerebrospinal fluid from patients with central nervous system tumors
Source: Acta Neuropathol Commun. 2024 Sep 17;12:151. doi: 10.1186/s40478-024-01846-4 (PMC11406943; doi:10.1186/s40478-024-01846-4)

# Supplementary Figure 1

a

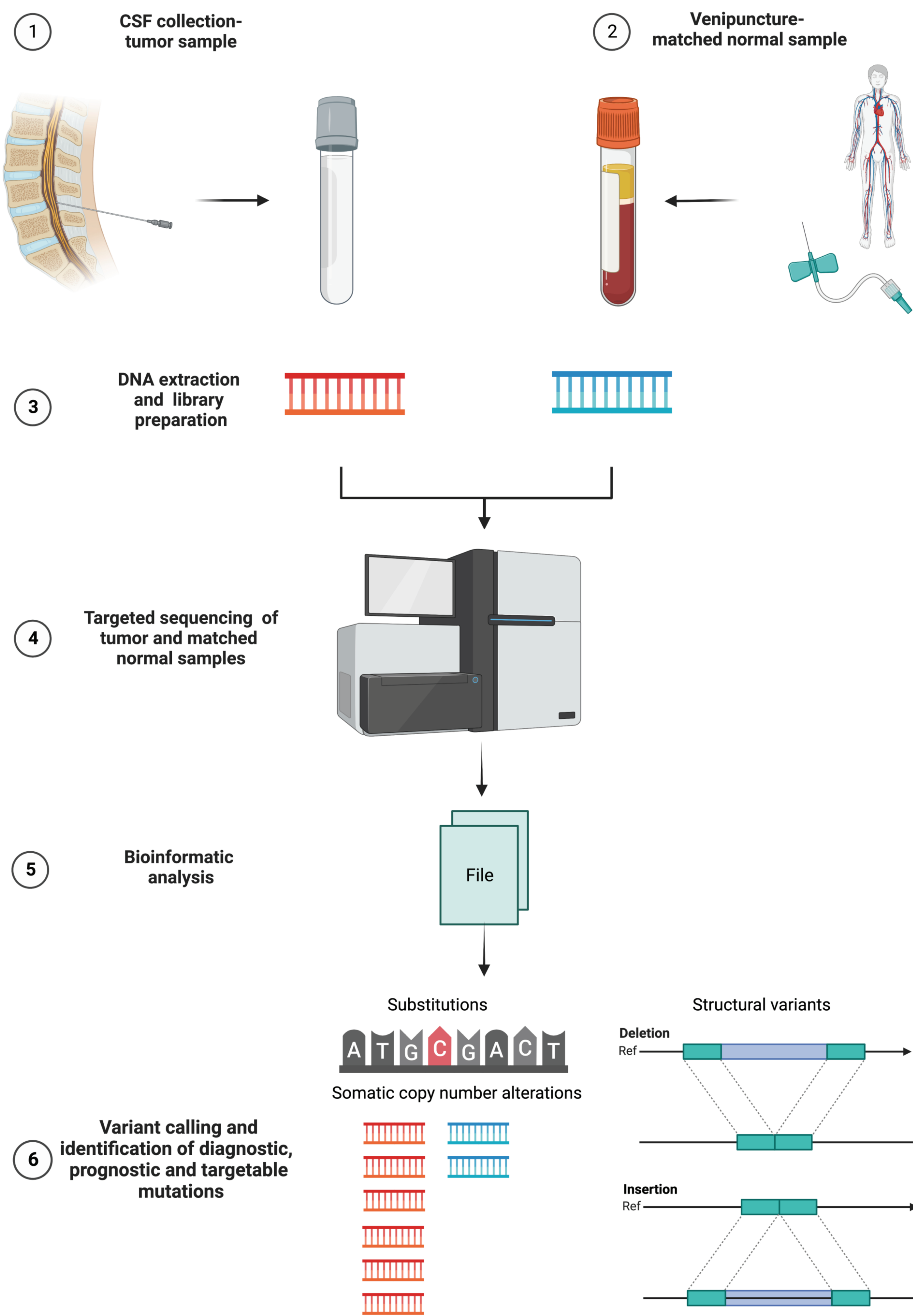

b

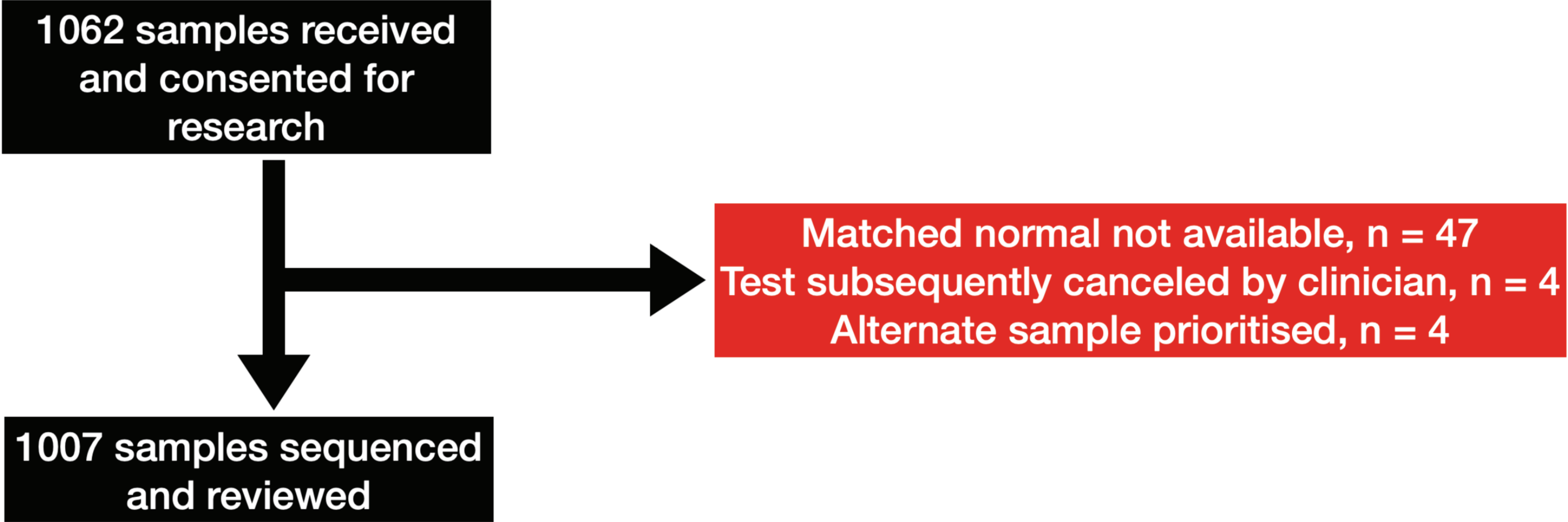

c

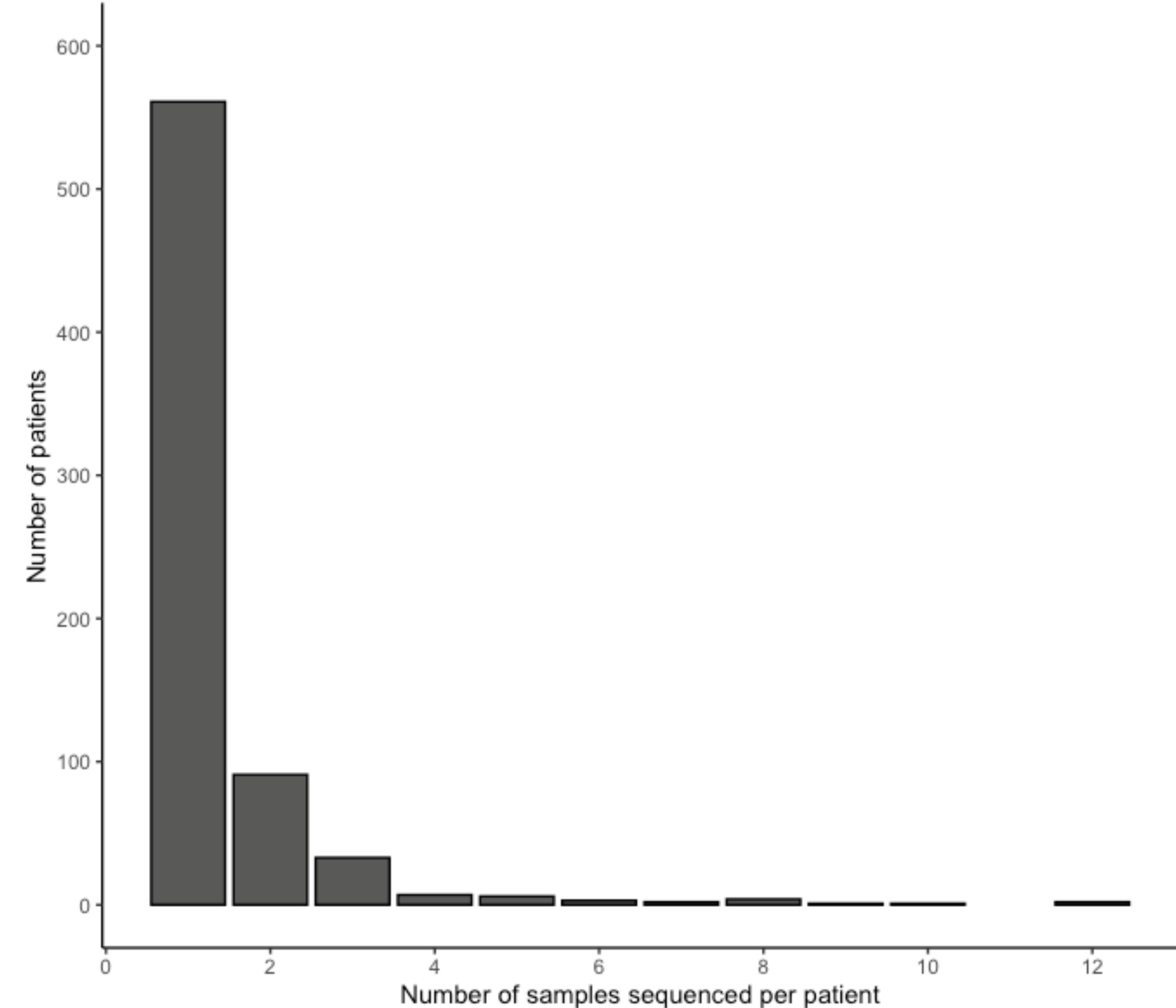

Supplement: Supplementary file 1 — Supplementary Material 1: Supplementary Figure 1. Study Overview.Summary of the MSK-IMPACT™ workflow for tumor matched normal sequencing of ctDNA from CSF. cfDNA from CSF and genomic DNA from normal blood are extracted and sequenced as Tumor:Normal pairs to facilitate the analysis of somatic variants including mutations, somatic copy number alterations and structural rearrangements.CONSORT diagram showing samples excluded from pre-analytic and genomic/clinical analyses in this study.Distribution of samples per patient across the overall cohort [file 40478_2024_1846_MOESM1_ESM.pdf]

Supplementary Figure 2

a

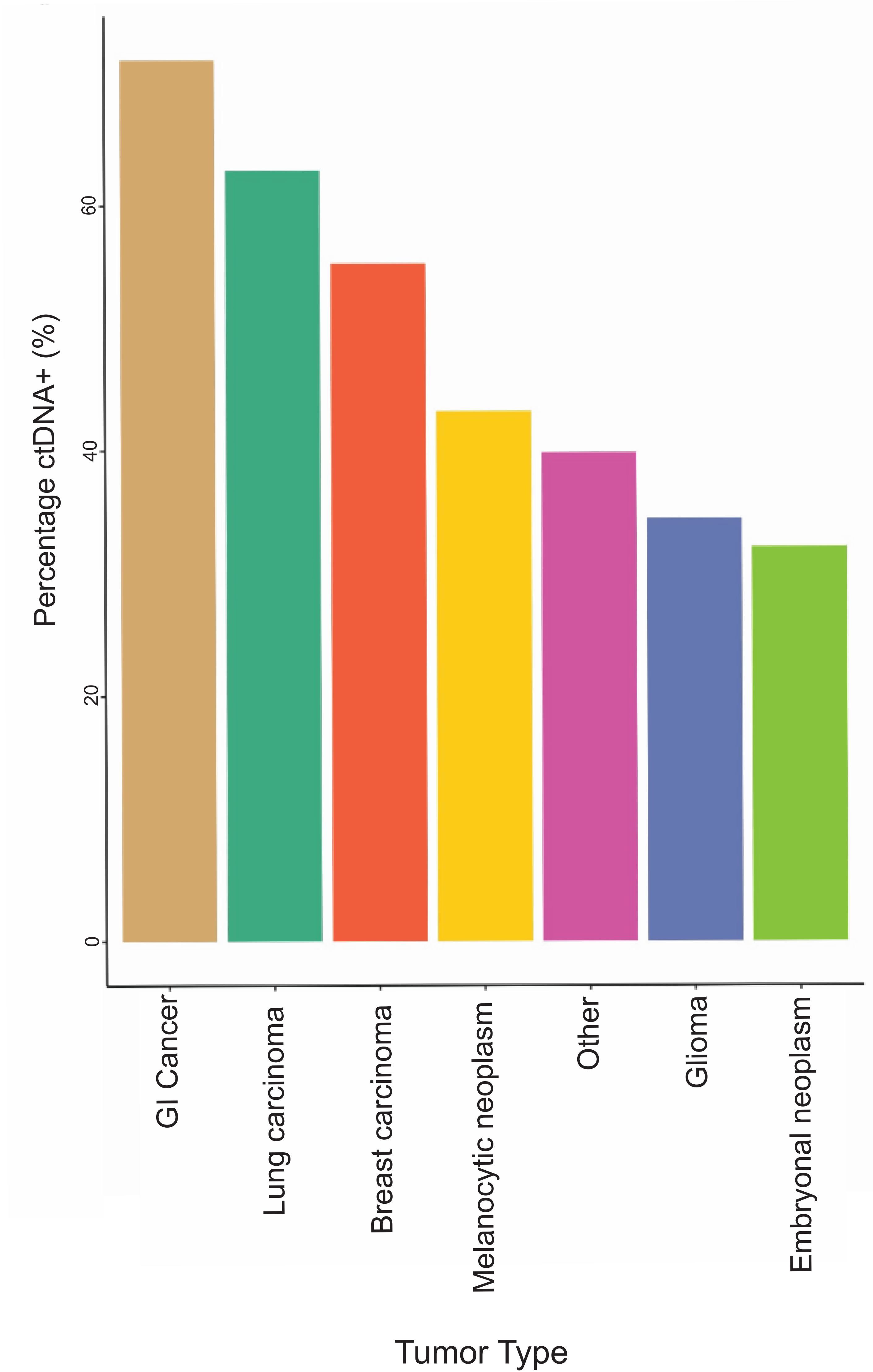

b

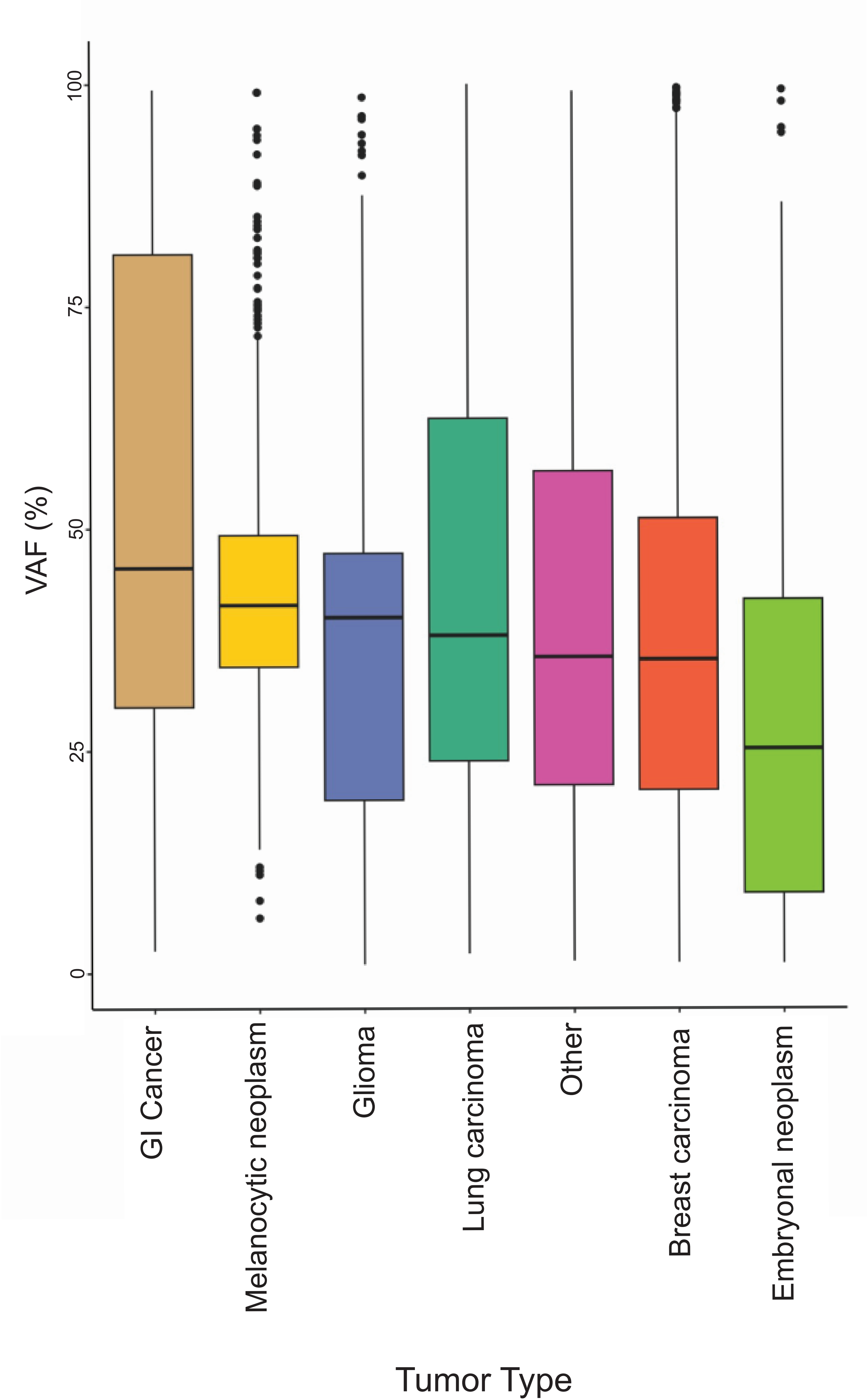

Supplement: Supplementary file 2 — Supplementary Material 2: Supplementary Figure 2. Variability of ctDNA-positivity and VAF across tumor types.Proportion of samples that are ctDNA+ by tumor type.Boxplots showing the distribution of VAFs across tumor types. Boxplots display the median, quartiles and range of values by tumor type [file 40478_2024_1846_MOESM2_ESM.pdf]

Supplementary Figure 3

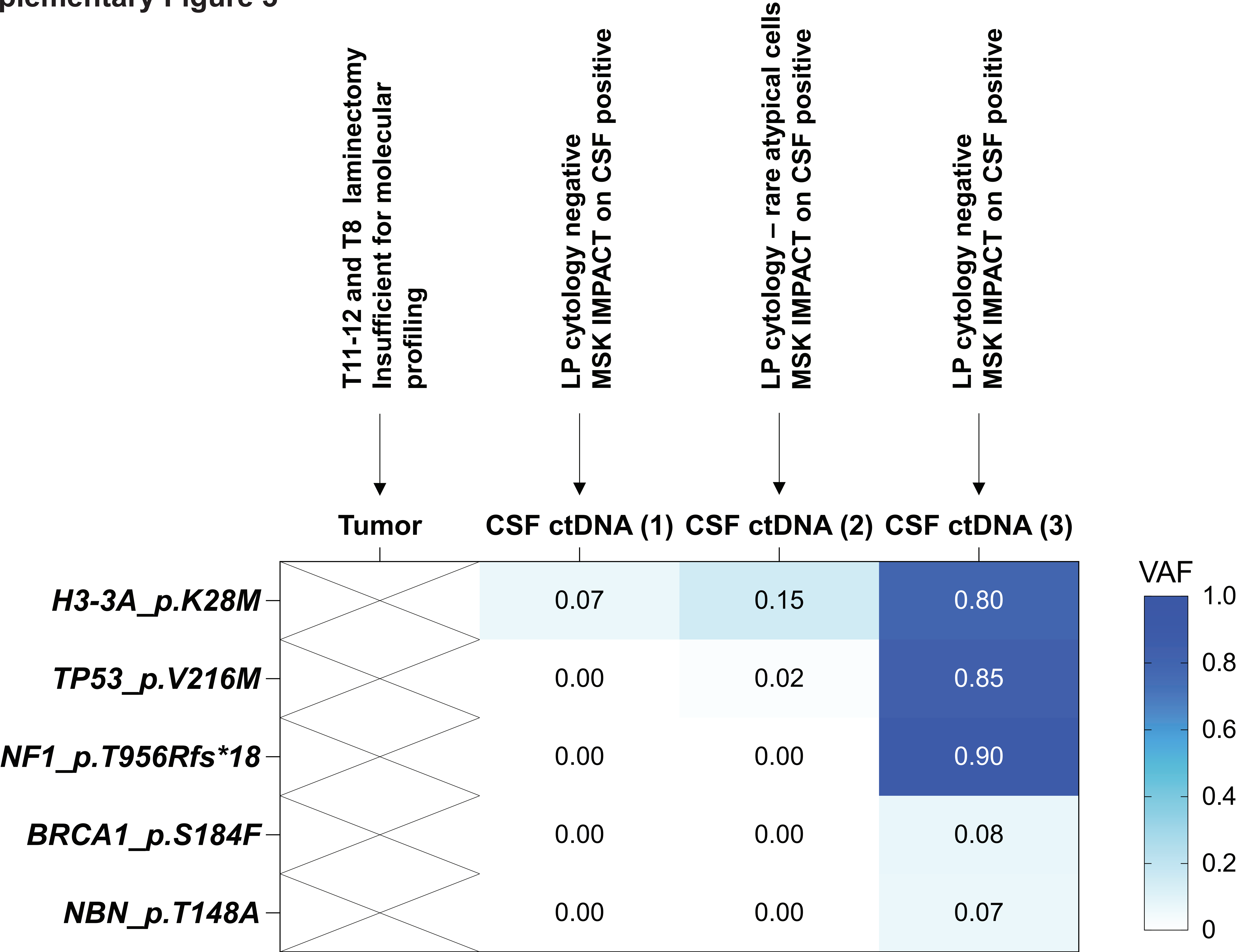

Supplement: Supplementary file 3 — Supplementary Material 3: Supplementary Figure 3. Tumor subclassification using CSF-ctDNA. A 31-year-old woman presented with an expansile T11-T12 intramedullary cord lesion. Biopsy was submitted for sequencing, but the tissue was insufficient for molecular analysis. CSF-ctDNA identified a histone mutation H3-3A p.K28M which, together with the clinical presentation, supported the diagnosis of diffuse midline gliomaaccording to the 2021 WHO Classification of Tumors of the Central Nervous System. Sequential monitoring by serial CSF sampling demonstrated increasing mutational load and VAF with disease progression. Throughout this time, cytologic assessment of the CSF remained negative [file 40478_2024_1846_MOESM3_ESM.pdf]

Supplementary Figure 4

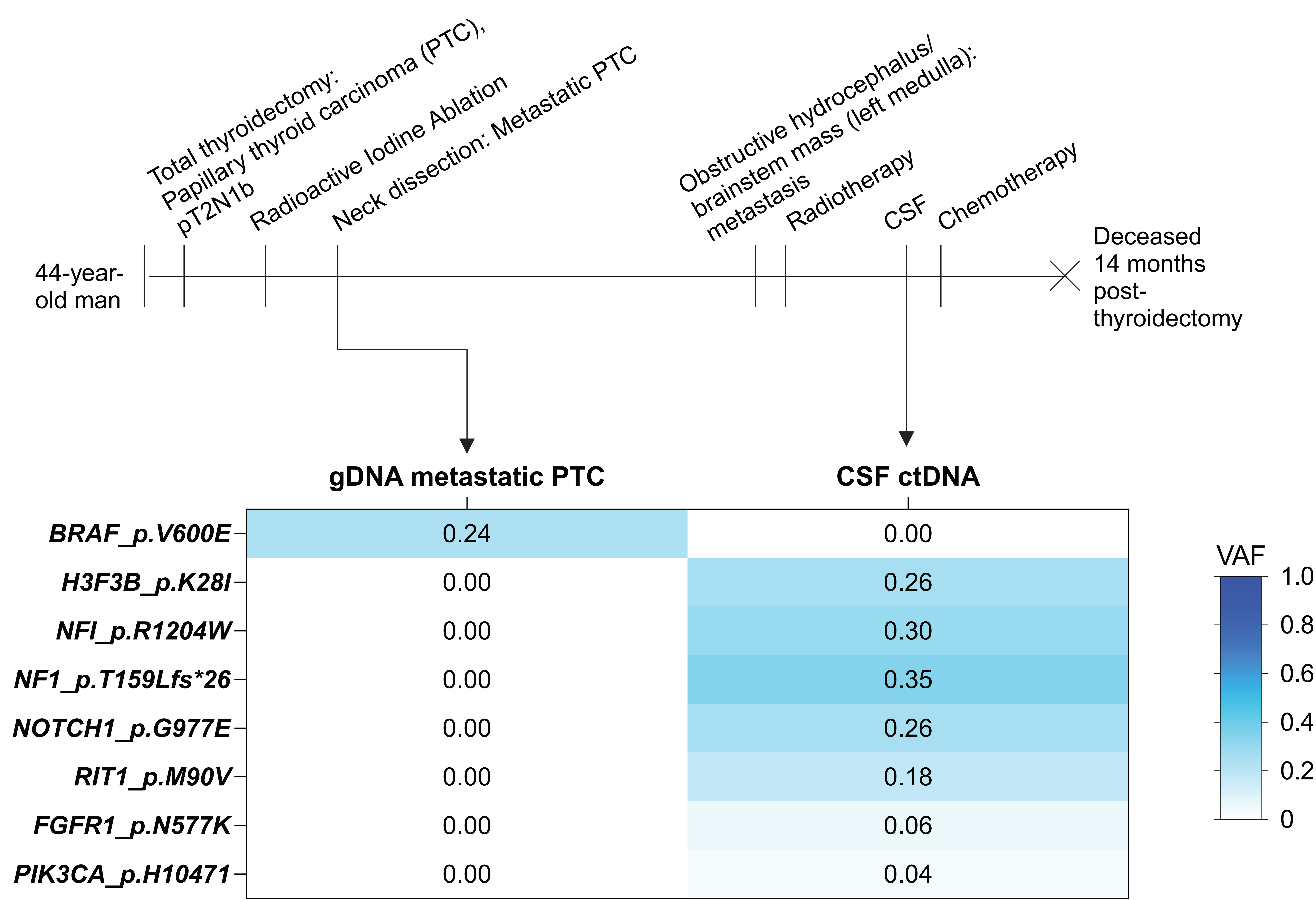

Supplement: Supplementary file 4 — Supplementary Material 4: Supplementary Figure 4. Diagnosis of primary tumor using CSF-ctDNA. This 44-year-old man presented to the hospital with a diagnosis of metastatic papillary thyroid carcinoma. Sequencing of genomic DNA from the tumor demonstrated a canonical BRAF p.V600E mutation. In subsequent months, the patient presents with a brain lesion suspected to represent a brain metastasis. CSF sequencing revealed a mutational profile that was distinct from and clonally unrelated to the papillary thyroid cancer, with a non-canonical histone mutation; these variants and the lack of the BRAF mutation indicated a second malignancyfor this patient [file 40478_2024_1846_MOESM4_ESM.pdf]

Supplementary Figure 5

a

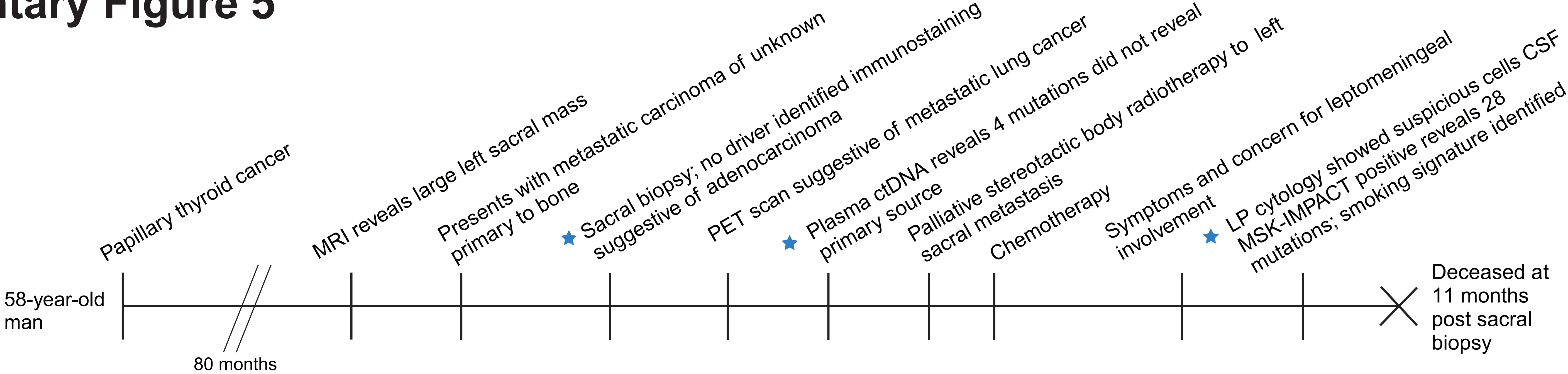

b

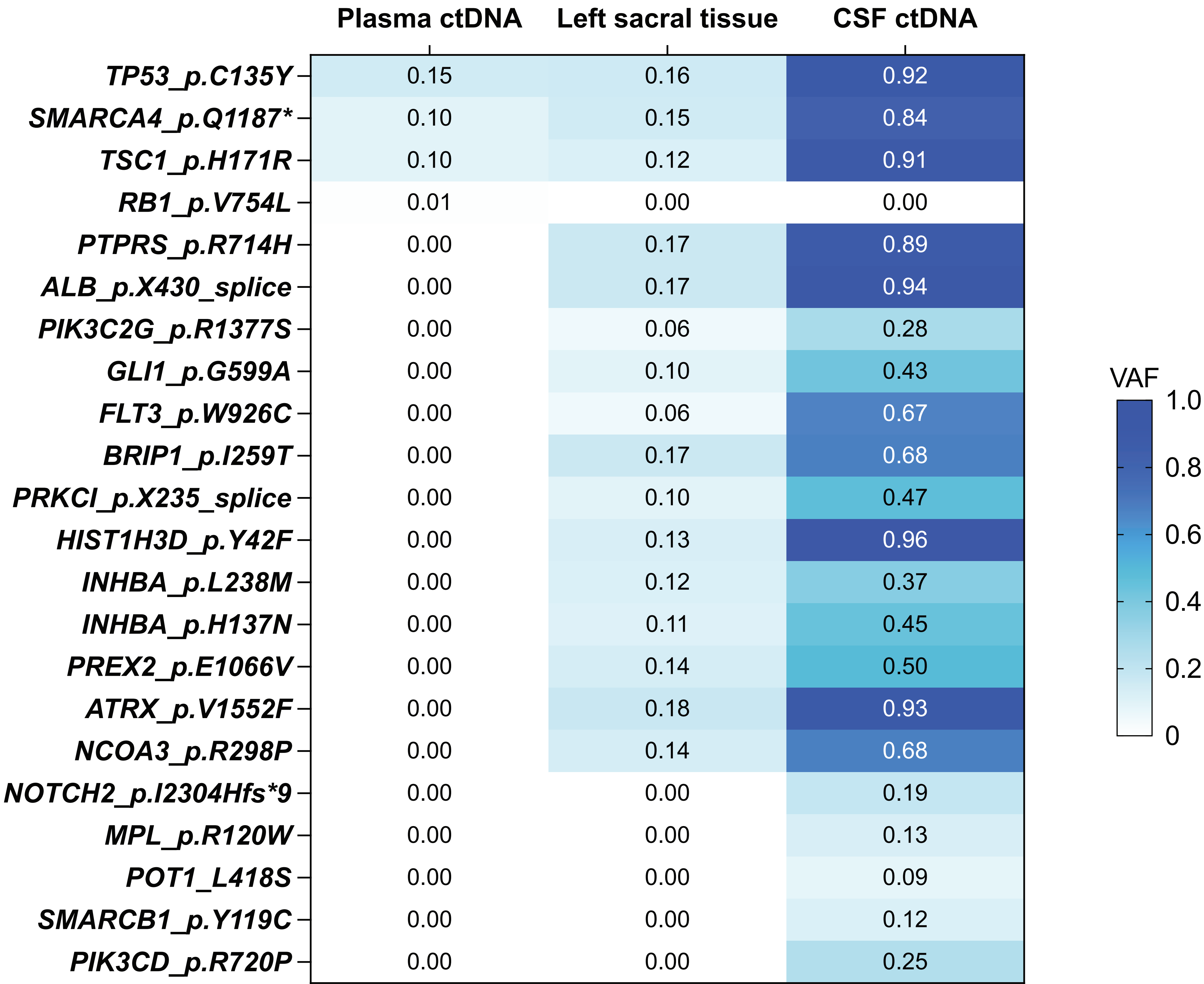

Smoking (65%)

c

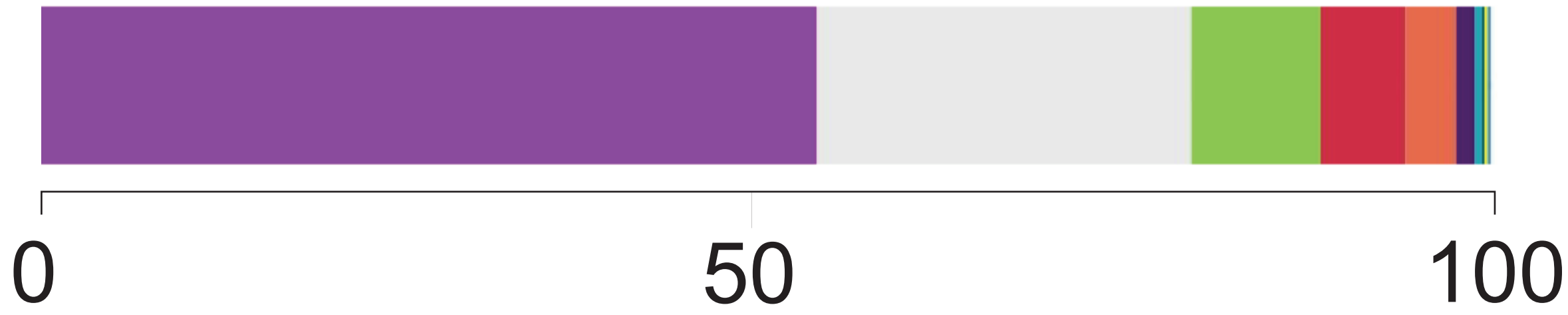

Supplement: Supplementary file 5 — Supplementary Material 5: Supplementary Figure 5. Determining clonal relatedness of CNS metastasis to a primary tumor using CSF-ctDNA. A 58-year-old man with history of thyroid carcinoma presented a metastatic bone lesion. Histological and immunohistochemical evaluation of the biopsy was non-conclusive, favoring an adenocarcinoma. Plasma testing was initiated and detected 4 mutations with non-specific profile. Sequencing of the sacral lesion did not reveal a driver alteration but pointed toward a metastasis from a lung primary site based on a weak smoking mutational signature. A few months later, the patient developed symptoms concerning for leptomeningeal involvement. Cytology showed rare, atypical cells; CSF-ctDNA identified 28 genetic alterations with high overlap to the sacral tumor profile and with a strong smoking-related mutational signature establishing clonal relatedness between. Together, this data established the diagnosis of metastatic NSCLC to the CNS.Outlines the sequence of events.Details of the sequencing results in order of availability. Note the marked difference in variant allele frequenciesidentified in the CSFcompared to the sacral biopsy. The table displays the mutations detected in each sample sequenced, along with the corresponding VAF’s, highlighted according to the color scale.Proportion of the genetic alterations identified in the tumor stratified by the type that would support independent mutational signatures. The estimated tumor mutation burdenfor this sample was 18.1 mutations per megabase. 65% of the mutations constituted C>A, G>T, CC>AA, and GG>TT transversions, supporting smoking-induced damage mutagenesis [file 40478_2024_1846_MOESM5_ESM.pdf]

Supplementary Figure 6

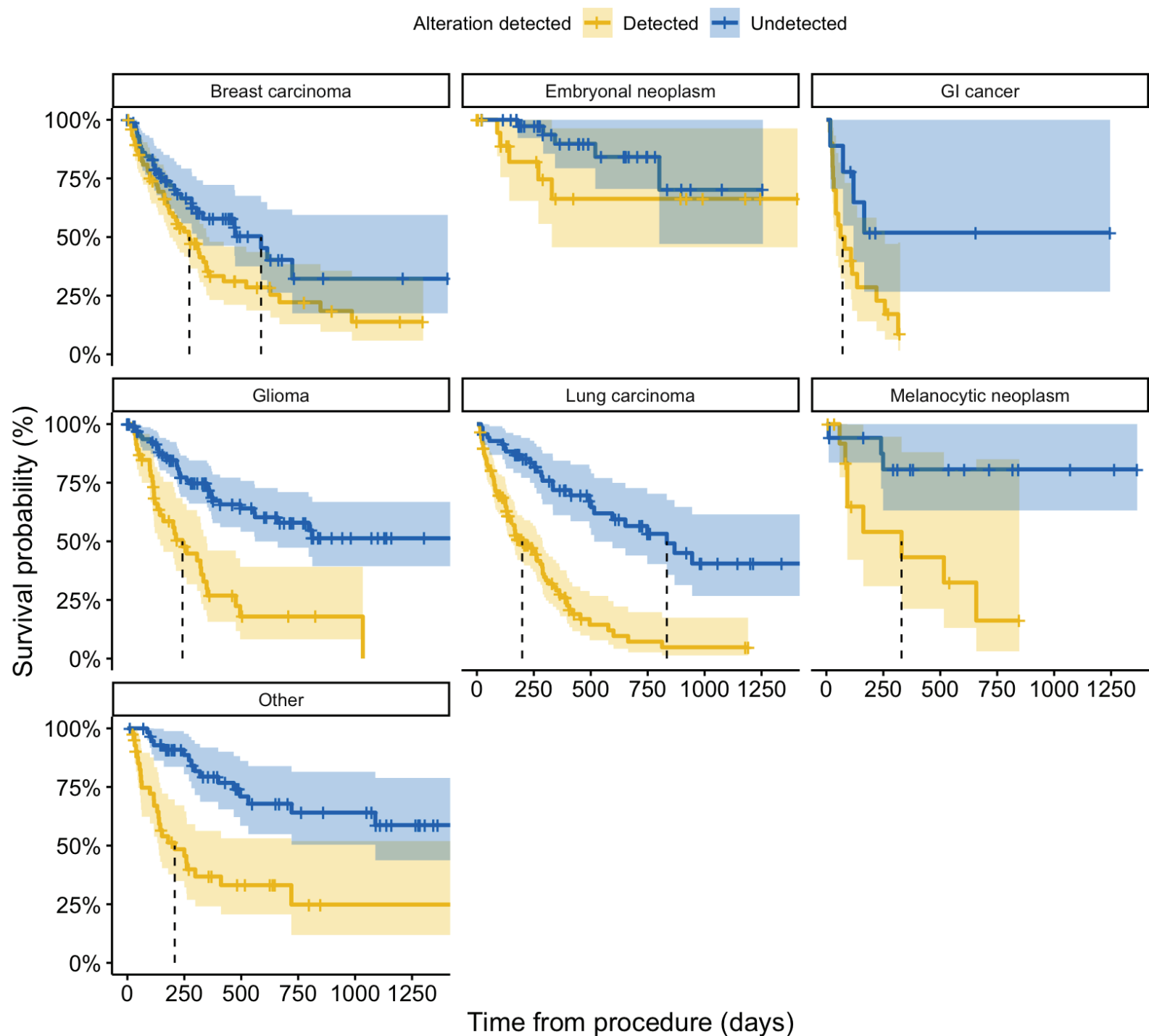

Supplement: Supplementary file 6 — Supplementary Material 6: Supplementary Figure 6. Detection of ctDNA shortens OS, irrespective of tumor type [file 40478_2024_1846_MOESM6_ESM.pdf]

Supplementary Figure 7

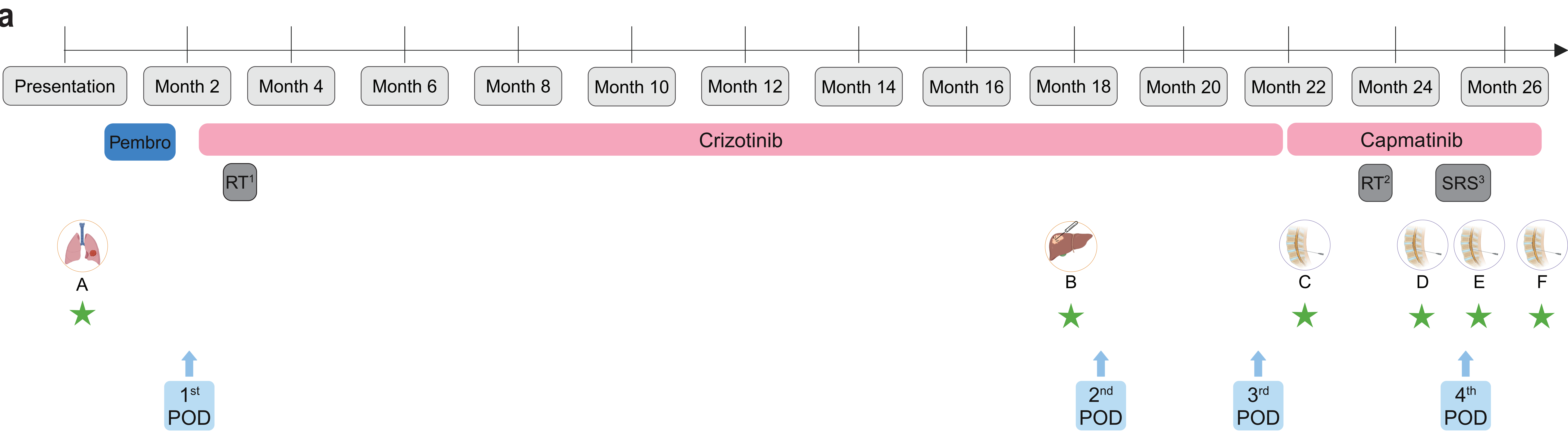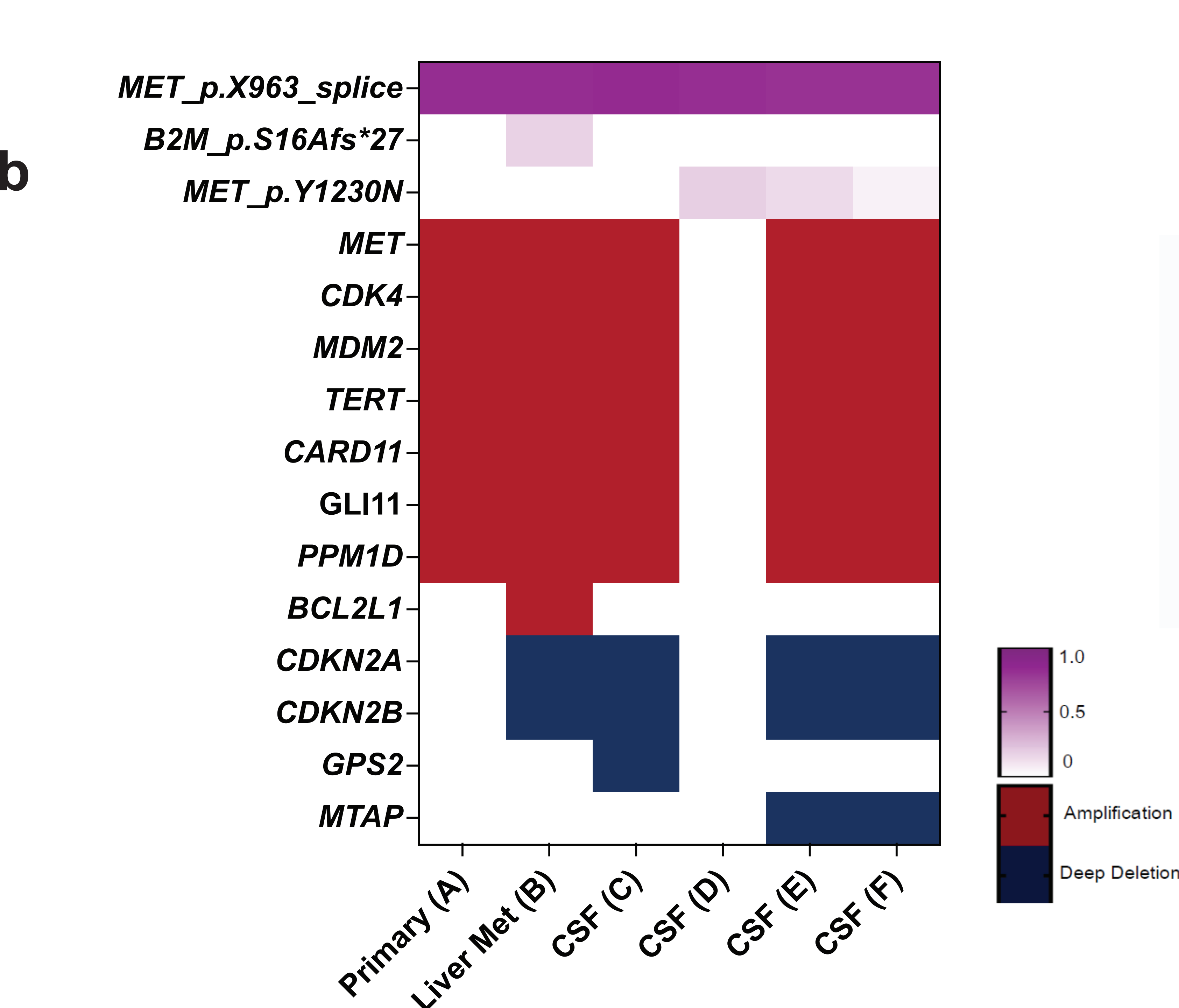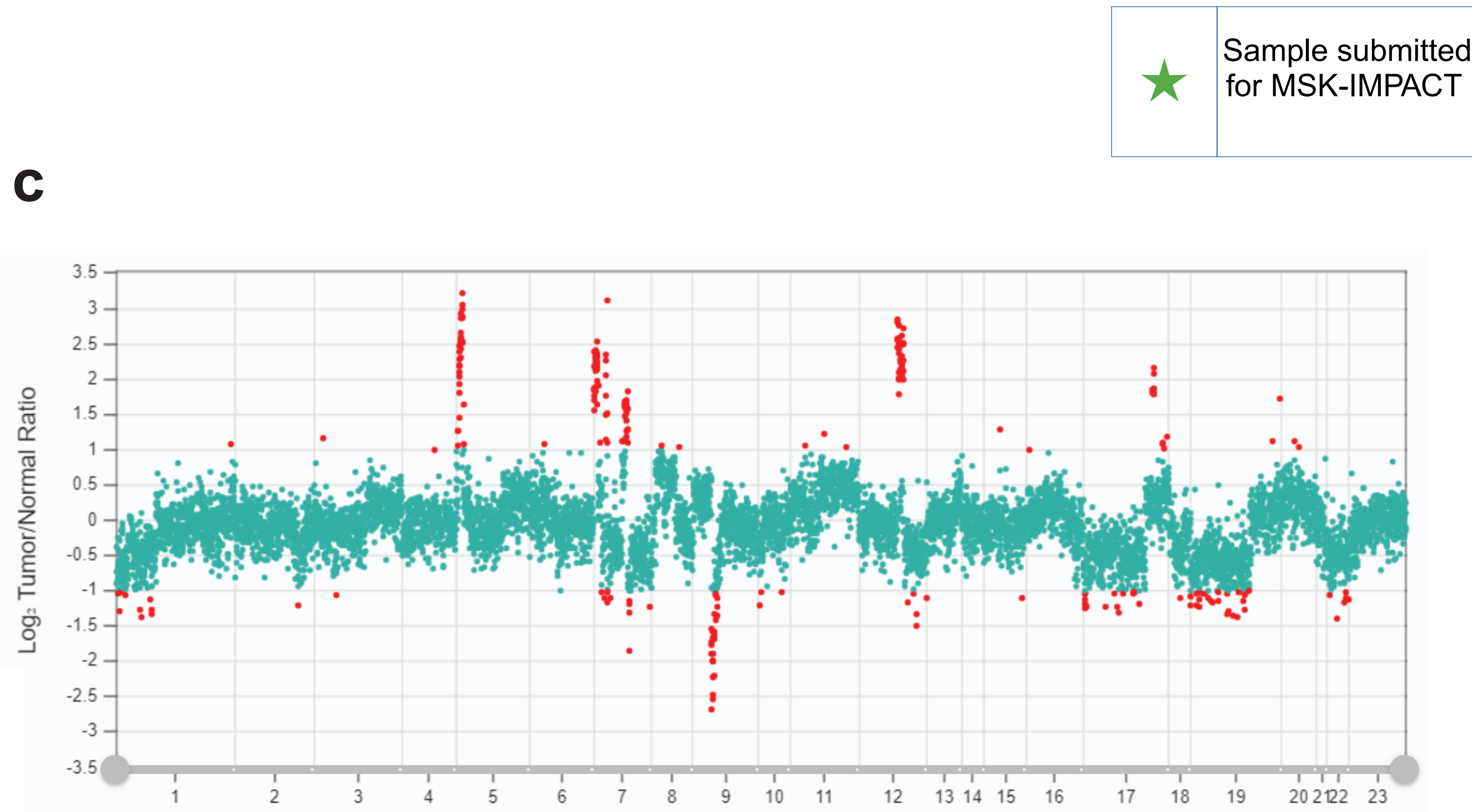

Supplement: Supplementary file 7 — Supplementary Material 7: Supplementary Figure 7. Monitoring of Drug Resistance using CSF-ctDNA. Shown is a representative case of a patient with lung adenocarcinoma with Met exon 14 skipping and concurrent MET amplification.Summary of the clinical course.sequencing results for the diagnostic tumor sample, liver metastasis and 4 CSF samples tested during the monitoring phase. Sequencing of the second CSF sample detected the emergence of a new MET alteration Y1230N while on crizotinib. The development of the MET mutations was only detected on the CSF but not in the metastatic lesion from liver. Given the documented leptomeningeal progression, the patient was transitioned to capmatinib.Demonstrates the copy number plot obtained from a CSF sample. Given the high ctDNA in CSF samples, high gains and deep losses can be readily observed. RT1 Palliative radiation to left femur. RT2 Palliative radiation therapy to metastatic cerebellar lesion, SRS3 Stereotactic radiosurgery to new metastatic cerebellar lesion and right trigeminal leptomeningeal lesion [file 40478_2024_1846_MOESM7_ESM.pdf]

# Supplementary Figure 8

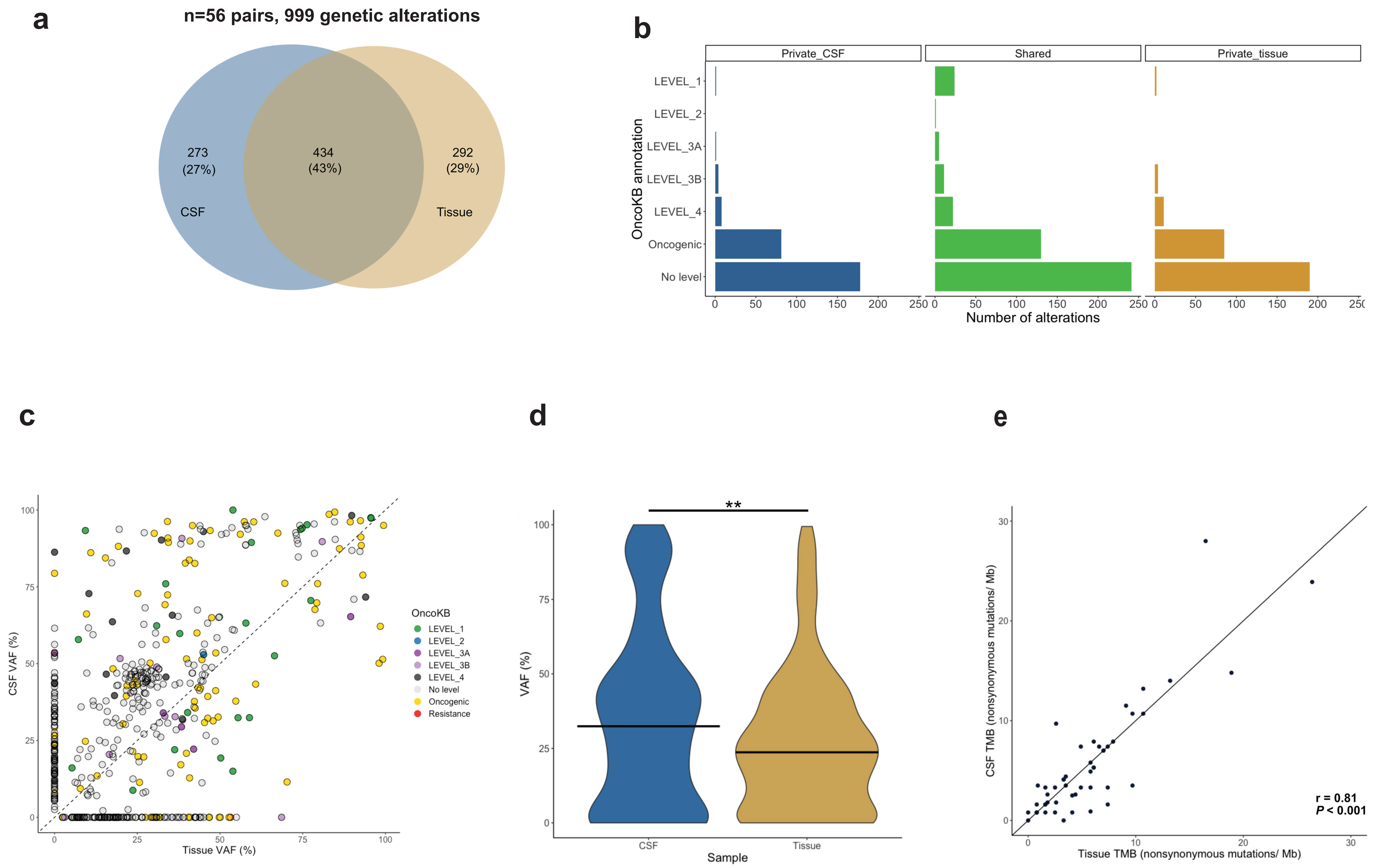

Supplement: Supplementary file 8 — Supplementary Material 8: Supplementary Figure 8. Comparison between Tumor DNA and CSF ctDNA. The data represents 56 tumor/CSF pairs from 55 patients who underwent collection of both samples within 90 days.Venn diagram showing the overlap of mutations between CSF and tumor. Overall, a total of 999 alterations were detected, 434/999alterations were shared between tumor and CSF, 273/999were private to the CSF and 292/999were private to the tumor biopsy.alterations are stratified based on level of actionability. The number of total mutations denoted on the x axis. When considering only those alterations with OncoKB levels of 1 to 3A, the frequency of shared CSF/tissue alterations was considerably higherthan private alterations to CSF or tissue.Comparison of mutational VAFs and their associated OncoKB levels between paired tissue and CSF samples.Comparison of VAFs for shared mutations reveals significantly higher levels in ctDNA from CSFcompared to the tumor tissue, despite routine enrichment by manual macro-dissection in solid tumor samples where necessary.Measurements of tumor mutation burdenin tumor tissueand CSFcorresponded closely with each other [file 40478_2024_1846_MOESM8_ESM.pdf]

# Supplementary Figure 9

a

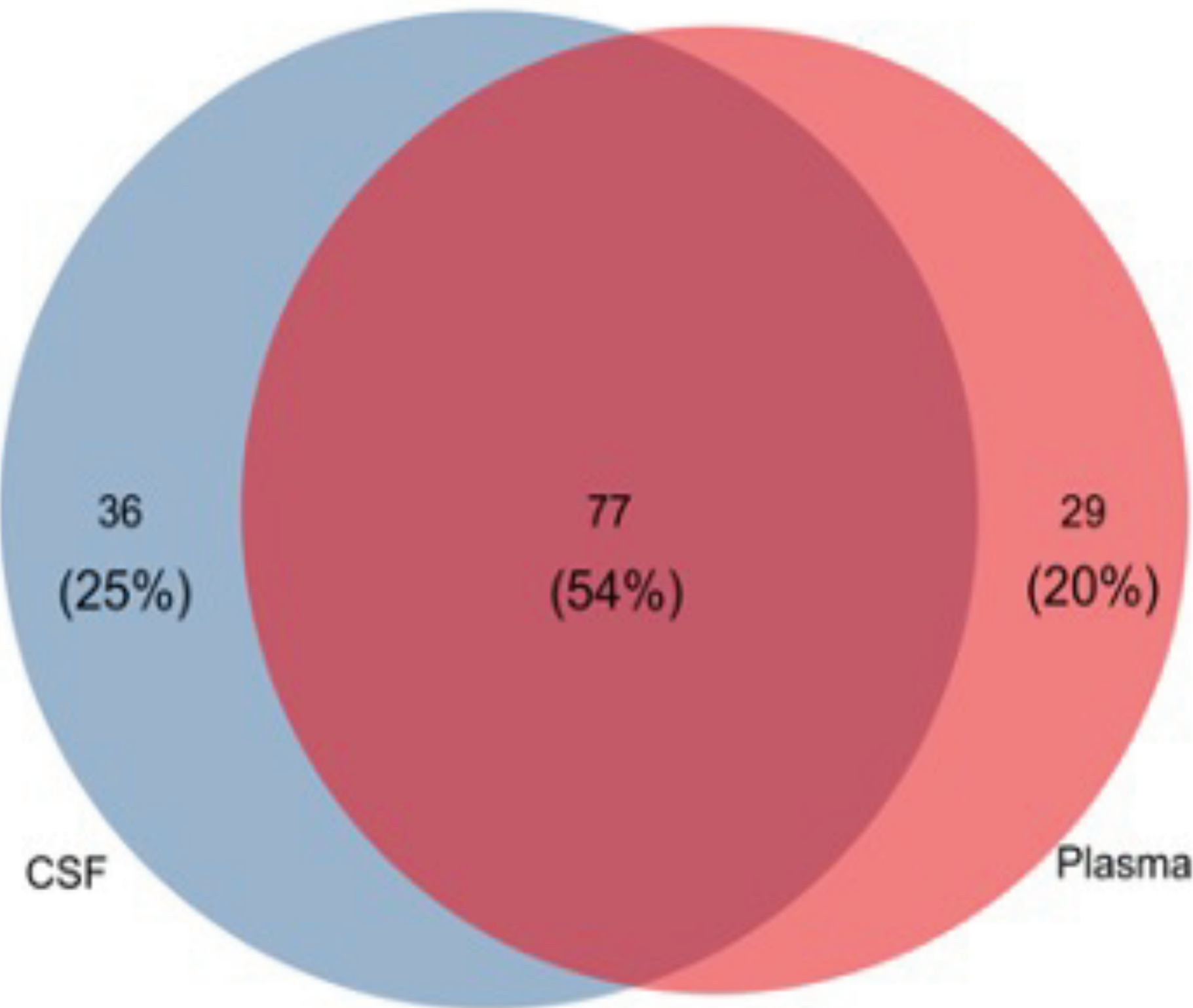

b

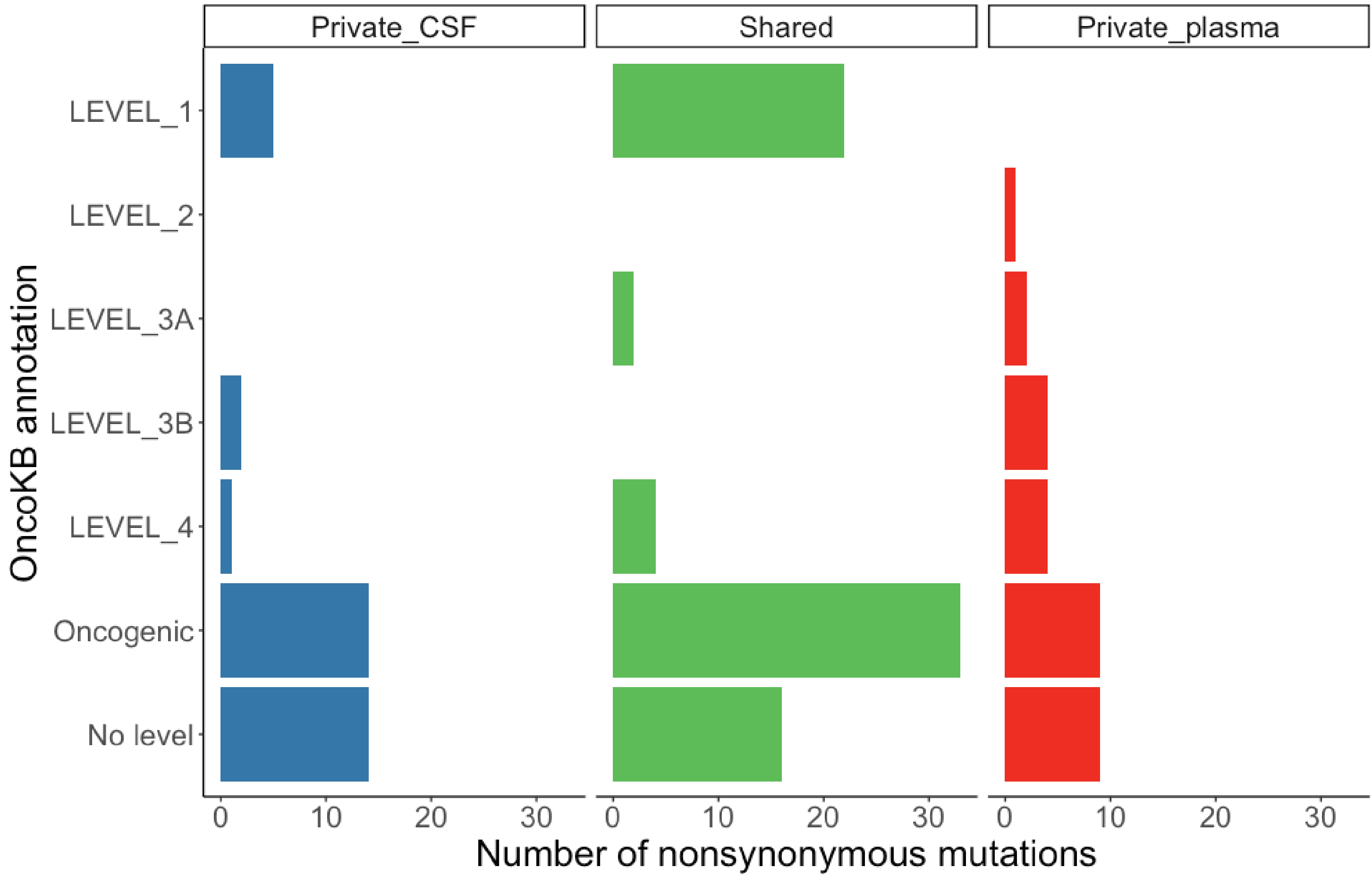

c

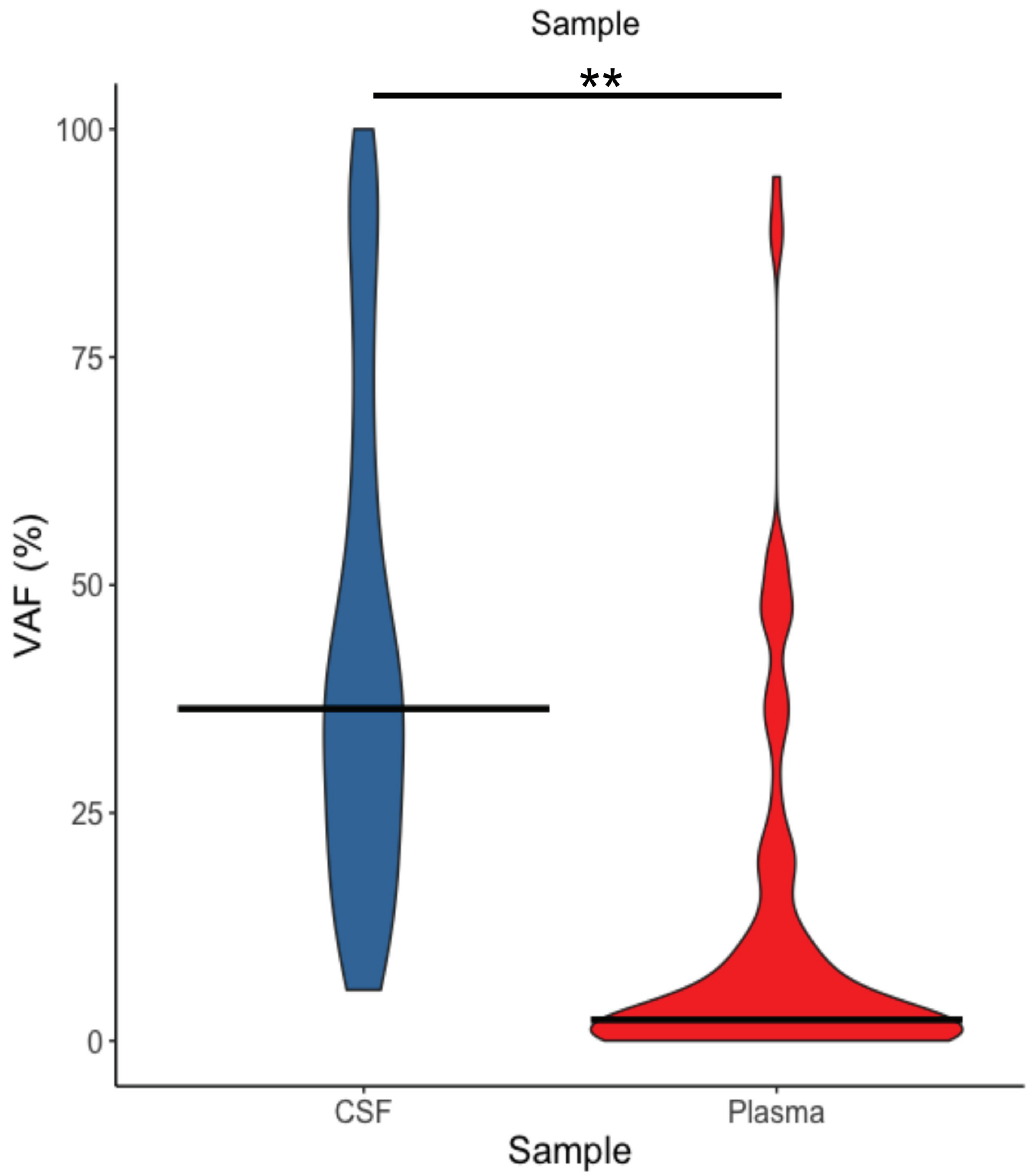

Supplement: Supplementary file 9 — Supplementary Figure 9. Comparison between Plasma ctDNA and CSF ctDNA. The data represents plasma ctDNA/CSF-ctDNA pairs from 31 patients/40 samples, breast carcinoma, GI cancerand the remaining 2 patients having CNS embryonal tumors) who underwent collection of both samples within 90 days. This analysis is restricted to the genomic regions covered by both assays.Venn diagram demonstrates that over half of the total alterations detected were shared between plasma and CSF.Alterations are stratified by level of actionability. All level 1 alterations are either shared or are private to the CSF.Comparison of VAFs for shared mutations reveals significantly higher levels in ctDNA from CSF Compared to the alterations identified in plasma. [file 40478_2024_1846_MOESM9_ESM.pdf]
