# Supplementary material for: Real-world experience with circulating tumor DNA in cerebrospinal fluid from patients with central nervous system tumors
Source: Acta Neuropathol Commun. 2024 Sep 17;12:151. doi: 10.1186/s40478-024-01846-4 (PMC11406943; doi:10.1186/s40478-024-01846-4)
Supplement: Supplementary file 10 — Supplementary Material 10. Supplementary tables. [file 40478_2024_1846_MOESM10_ESM.docx]

**Supplementary Table 1:** Patient demographics in the cohort. Age refers to the mean age of CSF collections.

|  | **Breast carcinoma,** N=150^1^ | **Embryonal neoplasm,** N=64^1^ | **GI cancer,** N=29^1^ | **Glioma,** N=148^1^ | **Lung carcinoma,** N=188^1^ | **Melanocytic neoplasm,** N=31^1^ | **Other,** N=101^1^ |
| --- | --- | --- | --- | --- | --- | --- | --- |
| **Age** | 54 (45, 61) | 7 (3, 14) | 49 (40, 60) | 45 (20, 62) | 63 (55, 70) | 52 (36, 62) | 55 (23, 67) |
| **Sex** |  |  |  |  |  |  |  |
| Female | 148 (99%) | 26 (41%) | 13 (45%) | 67 (45%) | 124 (66%) | 12 (39%) | 52 (51%) |
| Male | 2 (1.3%) | 38 (59%) | 16 (55%) | 81 (55%) | 64 (34%) | 19 (61%) | 49 (49%) |
| **Sample Count** |  |  |  |  |  |  |  |
| >1 | 32 (21%) | 29 (45%) | 3 (10%) | 24 (16%) | 41 (22%) | 9 (29%) | 12 (12%) |
| 1 | 118 (79%) | 35 (55%) | 26 (90%) | 124 (84%) | 147 (78%) | 22 (71%) | 89 (88%) |
| ^1^Median (IQR), n (%) | |  |  |  |  |  |  |

**Supplementary Table 2** Numbers at risk for survival curves showing that detection of ctDNA in CSF shortens overall survival

| **Survival Curve by Detection** | | | | | | | | |
| --- | --- | --- | --- | --- | --- | --- | --- | --- |
| **Undetected** | |  |  |  |  |  |  |  |
|  | T=0 | T=250 | T=500 | T=750 | T=1000 | T=1250 | T=1500 |  |
| At Risk | 372 | 228 | 121 | 68 | 36 | 16 | 0 |  |
| Events | 0 | 67 | 99 | 113 | 119 | 120 | 121 |  |
| **Detected** | |  |  |  |  |  |  |  |
|  | T=0 | T=250 | T=500 | T=750 | T=1000 | T=1250 | T=1500 |  |
| At Risk | 337 | 120 | 40 | 22 | 10 | 3 | 0 |  |
| Events | 0 | 157 | 206 | 215 | 218 | 219 | 219 |  |
|  |  |  |  |  |  |  |  |  |

**Supplementary Table 3.** Clinico-pathologic correlates of ctDNA positivity in metastatic lung cancer.

|  | all^a^ | CSF ctDNA (+) | CSF ctDNA (-) | OR (95% CI)^b^; p-value^b^ |
| --- | --- | --- | --- | --- |
|  | n=188 | n=118 | n=70 |  |
|  |  |  |  |  |
| Median (range) age (years) | 63 (23-83) | 62 (30-83) | 63 (23-83) | 1.01 (0.98-1.03); 0.49 |
| Male, n (%) | 65 (35) | 74 (63) | 21 (30) | Ref; Ref |
| Female, n (%) | 123 (65) | 44 (37) | 49 (70) | 0.68 (0.36-1.27); 0.22 |
| **Driver Alteration, n (%)** |  |  |  |  |
| EGFR | 99 (53) | 65 (55) | 34 (49) | 1.07 (0.41-2.79); 0.89 |
| KRAS | 30 (16) | 16 (14) | 14 (20) | 0.63 (0.20-1.92); 0.41 |
| ALK | 10 (5) | 4 (3) | 6 (9) | 0.31 (0.06-1.46); 0.14 |
| Other^c^ | 27 (12) | 19 (6) | 8 (11) | 1.43 (0.44-4.66); 0.55 |
| Driver negative | 22 (12) | 14 (12) | 8 (11) | Ref; Ref |
| **Disease Duration** |  |  |  |  |
| Median interval since diagnosis (days) | 764 | 751 | 821 | 1.0003 (0.9999-1.0007); 0.11 |
| **Type of CNS Involvement, n (%)** |  |  |  |  |
| Parenchymal disease only | 71 (38) | 16 (14) | 55 (79) | Ref; Ref |
| Leptomeningeal disease only | 24 (13) | 23 (20) | 1 (1) | 38.84 (12.83-117.54); <0.0001 |
| Both parenchymal and leptomeningeal disease | 94 (50) | 79 (67) | 15 (21) | 20.17 (9.65-42.16); <0.0001 |

^a^Unique patients only. Collection closest to diagnosis taken if more than one entry for a patient.

^b^Multiple entries per patient included in modeling.

^c^*ERBB2*, *BRAF*, *MET*, *RET*, *ROS1*, *EGFR/ERBB2*, *EGFR/MET*

**Supplementary Table 4:** Comparisons of CSF volume, DNA metrics and sample coverages between samples that are ctDNA+ versus ctDNA- across 1007 samples

| **Variable** | **ctDNA+**, N=489^1^ | **ctDNA-**, N=518^1^ | **p-value**^2^ |
| --- | --- | --- | --- |
| **CSF volume (ml)** | 5.5 (5.0, 9.0) | 5.5 (4.0, 9.0) | <0.001 |
| **DNA yield (ng)** | 1.7 (0.4, 6.4) | 0.1 (0.1, 0.3) | <0.001 |
| **DNA concentration (ng/ml)** | 0.3 (0.1, 1.3) | 0.0 (0.0. 0.1) | <0.001 |
| **% cfDNA** | 49.5 (35.8, 62.6) | 25.1 (18.0, 36.5) | <0.001 |
| **Median coverage (X)** | 247.0 (103.0, 466.0) | 17.0 (9.0, 47.0) | <0.001 |
| ^1^Median (IQR) |  |  |  |
| ^2^Wilcoxon rank sum test |  |  |  |
